# National youth sedentary behavior and physical activity daily patterns using latent class analysis applied to accelerometry

**DOI:** 10.1186/s12966-016-0382-x

**Published:** 2016-05-03

**Authors:** Kelly R. Evenson, Fang Wen, Derek Hales, Amy H. Herring

**Affiliations:** Department of Epidemiology at the Gillings School of Global Public Health, Center for Health Promotion and Disease Prevention, University of North Carolina, 137 East Franklin Street, Suite 306, Chapel Hill, NC 27514 USA; Department of Epidemiology at the Gillings School of Global Public Health, University of North Carolina, Chapel Hill, NC USA; Department of Nutrition at the Gillings School of Global Public Health, University of North Carolina, Chapel Hill, NC USA; Department of Biostatistics at the Gillings School of Global Public Health, Carolina Population Center, University of North Carolina, Chapel Hill, NC USA

**Keywords:** Accelerometry, Children, Cluster analysis, Latent class analysis, Surveillance

## Abstract

**Background:**

Applying latent class analysis (LCA) to accelerometry can help elucidated underlying patterns. This study described the patterns of accelerometer-determined sedentary behavior and physical activity among youth by applying LCA to a nationally representative United States (US) sample.

**Methods:**

Using 2003–2006 National Health and Nutrition Examination Survey data, 3998 youths 6–17 years wore an ActiGraph 7164 accelerometer for one week, providing > =3 days of wear for > =8 h/day from 6:00 am-midnight. Cutpoints defined sedentary behavior (<100 counts/minute), light activity (100–2295 counts/minute), moderate to vigorous physical activity (MVPA; > = 2296 counts/minute), and vigorous activity (> = 4012 counts/minute). To account for wear time differences, outcomes were expressed as percent of day in a given intensity. LCA was used to classify daily (Monday through Sunday) patterns of average counts/minute, sedentary behavior, light activity, MVPA, and vigorous activity separately. The latent classes were explored overall and by age (6–11, 12–14, 15–17 years), gender, and whether or not youth attended school during measurement. Estimates were weighted to account for the sampling frame.

**Results:**

For average counts/minute/day, four classes emerged from least to most active: 40.9 % of population (mean 323.5 counts/minute/day), 40.3 % (559.6 counts/minute/day), 16.5 % (810.0 counts/minute/day), and 2.3 % (1132.9 counts/minute/day). For percent of sedentary behavior, four classes emerged: 13.5 % of population (mean 544.6 min/day), 30.1 % (455.1 min/day), 38.5 % (357.7 min/day), and 18.0 % (259.2 min/day). For percent of light activity, four classes emerged: 12.3 % of population (mean 222.6 min/day), 29.3 % (301.7 min/day), 41.8 % (384.0 min/day), and 16.6 % (455.5 min/day). For percent of MVPA, four classes emerged: 59.9 % of population (mean 25.0 min/day), 33.3 % (60.9 min/day), 3.1 % (89.0 min/day), and 3.6 % (109.3 min/day). For percent of vigorous activity, three classes emerged: 76.8 % of population (mean 7.1 min/day), 18.5 % (23.9 min/day), and 4.7 % (47.4 min/day). Classes were developed by age, gender, and school attendance since some patterns differed when stratifying by these factors.

**Conclusion:**

The models supported patterns for average intensity, sedentary behavior, light activity, MVPA, and vigorous activity. These latent class derived patterns can be used in other youth studies to explore correlates or outcomes and to target sedentary behavior or physical activity interventions.

**Electronic supplementary material:**

The online version of this article (doi:10.1186/s12966-016-0382-x) contains supplementary material, which is available to authorized users.

## Background

In 2010, the World Health Organization recommended that youth 5–17 years should accumulate at least 60 min of moderate to vigorous physical activity (MVPA) daily, with amounts great than 60 min/day conferring additional health benefits [1]. They also recommended that vigorous intensity activities should be incorporated at least 3 times/week. This recommendation was similar to the 2008 United States (US) Physical Activity Guidelines for Americans that recommended similar amounts of MVPA and vigorous activity [2].

Accelerometry is increasingly being used to conduct surveillance for physical activity and sedentary behavior among youth. This type of measure can provide information at very small increments of time called epochs. Usually, studies aggregate the information provided by the accelerometer to a daily or weekly average. While this aggregation is useful, and allows for exploration into whether or not a participant met physical activity guidelines, it can mask underlying *patterns* of behavior throughout the week.

The application of latent class analysis (LCA) is emerging as a statistical method to describe patterns of physical activity and sedentary behavior using accelerometry. For example, it has been used in national studies of adults to describe accelerometer-assessed patterns of physical activity and sedentary behavior [3, 4] and to use those patterns to explore correlates [5]. Among youth, one study of adolescents derived latent classes using self-reported physical activity and sedentary behavior [6, 7], and another study of 6th to 11th graders combined self-reported physical activity with accelerometer-assessed MVPA to create latent classes [8]. The derivation of latent class assignments among youth can subsequently be used to explore correlates or outcomes with those classifications, and to target sedentary behavior or physical activity interventions by those patterns.

While LCA has been applied to measures of physical activity among youth, the studies reviewed did not include younger ages. No youth studies were found that focused on accelerometry only to derive detailed patterns or that applied LCA to accelerometer-assessed sedentary behavior. The purpose of this study was to identify classes of sedentary behavior and physical activity patterns among a national sample of US youth. We explored whether these patterns differed by age, gender, and whether or not they were attending school at the time of measurement.

## Methods

The National Health and Nutrition Examination Survey (NHANES) data used in this study were obtained during 2003–2006, the most recent publicly available data with accelerometry. Participants, or if applicable due to age their parents or guardians, provided informed consent before the interview or any measurements were taken. The consenting documents are available for the 2003–2004 [9] and 2005–2006 [10] cohorts. Additionally, this research was approved by the University of North Carolina Institutional Review Board.

## Accelerometry measurement

Youth ages 6 and older were asked to wear the ActiGraph accelerometer (model #AM7164) on their hip for seven consecutive days during waking hours and outside of any water-based activities. Beginning at midnight on the day following the clinic visit, the accelerometer recorded 1-minute epochs of analog acceleration and converted it to a digital signal. The 24-hour data were reduced to recordings from 6:00 am to midnight. Nonwear was defined from the accelerometer by 1-minute time intervals with consecutive zero counts for at least a 90-minute time window (window 1), allowing short time intervals with nonzero counts lasting up to 2 min (allowance interval) if no counts are detected during both the 30 min (window 2) upstream and downstream from that interval. Any nonzero counts except the allowed short intervals were considered wearing time. This algorithm was developed on youths and adults, with evidence for validity [11].

We characterized average intensity of physical activity using average counts/minute. Then, using cutpoints from a calibration study [12] shown to be useful for ages 5 to 15 years [13], we defined various intensities of physical activity including sedentary, light, moderate, vigorous, and MVPA. Since the cutpoints were derived in 15-second epochs, we translated them as follows:

- Sedentary behavior: 0–25 counts/15-seconds to 0–99 counts/minute
- Light: 26–573 counts/15-seconds to 100–2295 counts/minute
- Moderate: 574–1002 counts/15-seconds to 2296–4011 counts/minute
- Vigorous: > = 1003 counts/15-seconds to > =4012 counts/minute
- MVPA: > = 574 counts/15-seconds to > =2296 counts/minute

Because the ActiGraph processing aggregates counts, and cutpoints are estimated based on steady state activities, the longer epoch should approximate the shorter one. Sedentary behavior is defined as waking activities performed while sitting or in a reclining posture [14]. Unfortunately the ActiGraph used for this study cannot distinguish sedentary behavior from standing without moving. Thus the term “sedentary” used in this paper includes some standing without moving.

## Other measures

Self-reported sociodemographic measures used in this report included age, gender, race/ethnicity (Non-Hispanic White, Non-Hispanic Black, Hispanic, other), and whether or not they were attending school at the time of measurement. Out of school could indicate they were on vacation or between grades in school.

## Statistical methods

The sample was limited to those age 6 to 17 years (*n* = 5607), who participated in the accelerometer portion of NHANES during 2003–2006 (*n* = 5030). We further excluded 355 participants whose accelerometer was not in calibration or was faulty upon return (i.e., recording no counts) and 677 who did not provide at least 3 days of accelerometer wear for > =8 h/day (from 6:00 am to midnight) over a seven-day period. This left a final sample size of 3998 in which 8.7 % had three days of wear (*n* = 349), 12.6 % four days (*n* = 503), 17.9 % five days (*n* = 717), 27.5 % six days (*n* = 1099), and 33.3 % seven days (*n* = 1330). Intraclass correlation coefficients were calculated for average counts/minute, percent light, percent MVPA, percent vigorous, and percent sedentary behavior among the full sample (*n* = 3998). The intraclass correlation coefficients ranged from 0.69 to 0.75 for 3 days, except for vigorous activity which produced the lowest value (0.64). Due to the similarities to more days of wear, we chose > =3 days for > =8 hours/day of wear as the minimum wear criteria. Following this, we explored day-level outliers among the accelerometry measures by exploring descriptive statistics and box plots. Four participants with extreme outliers on a given day were removed, but these participants were still retained in the overall sample since they provided > =3 days of accelerometry data even with the day removed.

To account for the differential probability of selection, all percents and means were weighted to the 2000 census using the 4-year sample weights provided by NHANES. The data were nested (i.e., screener, household interview, examination), such that non-response and post-stratification adjustments were applied.

Using LCA, we used 3 to 7 adherent days from the participant’s accelerometry to determine classes, or natural groupings, of participants who tended to accumulate their physical activity or sedentary behavior in a similar daily pattern. The derived classes were among participants who shared similar means, separately calculated for the following weighted indicators:

- counts per minute per day,
- percent of sedentary behavior out of total wearing time per day,
- percent of light activity out of total wearing time per day,
- percent of MVPA out of total wearing time per day, and
- percent of vigorous activity out of total wearing time per day.

The LCA was performed using MPlus (version 7.11) [15], which allowed for the complex survey design in conjunction with the modeling. Mixture modelling was applied to describe the relationship between up to 7 adherent days of accelerometry and the categorical latent variable using a set of linear regression equations. Although we had information on day of the week of measurement, we did not have access to the time ordering of the measures (e.g., one participant might start the 7 day window on Thursday, so that Wednesday’s and Thursday’s data were collected one week apart). Our latent class models do not make assumptions about proximity of weekdays, but rather control for day of the week without the assumption that Wednesday and Thursday’s measures fall on adjacent days.

Several criteria were used to guide the final number of classes for each sedentary behavior or physical activity variable which included:

1. the bootstrap likelihood ratio test, which compared the fit of k classes to (k-1) classes,
2. sample size of the classes, and
3. substantive knowledge, including a practical interpretation of what each class represented, along with visual inspection, to ensure that the classes were sufficiently separated from each other (entropy).

For each variable, we began with a 2- or 3-class model and continued up to 7 classes. Beyond this point the sample sizes of the most active and most sedentary classes became too small. Each participant was assigned to one class based on the highest posterior class membership probability. In addition, a sensitivity analysis was conducted among those who contributed two adherent weekend days and therefore one or more adherent weekdays (*n* = 2215) to explore whether missing weekends days impacted the results. The patterns for average intensity, sedentary behavior, light activity, MVPA, and vigorous activity were similar to those in the overall sample (*n* = 3998) and therefore not presented.

To test measurement invariance across age group, gender, and school characteristics, a multi-group LCA analysis was implemented using the same number of latent classes developed on the same physical activity measure on the full sample. Two multi-group LCA models were built: an unrestricted model and a restricted model where model parameters of day-to-day accelerometer measures were restricted to be equal across groups. Likelihood ratio chi-square tests were performed by comparing model fit from the two models (unrestricted vs. restricted). A significant p value indicated that a LCA model should be conducted by groups. Using SAS ® release 9.3 (Cary, North Carolina), classes were explored using weighted means of each variable by day of the week and for the overall results by accelerometer wear time.

Agreement between the overall assignment and the stratified assignment were compared using percent agreement and weighted kappa coefficients (e.g., comparing the assignment among males from the overall LCA to the assignment from the male-only LCA). Weighted kappa coefficients were interpreted as 0–<0.2 poor, 0.2- < 0.4 fair, 0.4- < 0.6 moderate, 0.6- < 0.8 substantial, and 0.8- < 1.0 almost perfect [16], with a lower coefficient indicating a greater need for the stratified assignment.

## Results

The sample comprised 3998 youth 6 to 17 years, distributed relatively evenly by age group (39.7 % 6–11 years, 31.2 % 12–14 years, 29.1 % 15–17 years) and gender (49.8 % female, 50.2 % male). More youth reported attending school at the time of the measurement (77.9 %) compared to not being in school (22.1 %), while 307 were missing on this measure. Youth 6 to 17 years were classified into 4 classes for average counts/minute/day and percent of time in sedentary behavior, light activity, and MVPA and 3 classes for percent of time in vigorous activity. The bootstrap likelihood ratio test to compare k to (k-1) classes was <0.001 in all cases.

For each latent class, we explored accelerometer wear time overall and by day within each derived class. For all variables, weighted mean weekly accelerometer wear time ranged from 12.9 to 13.3 h/day by derived class (data not shown). Mean wear was slightly longer for classes with more sedentary behavior (13.2 h/day class 4) compared to less sedentary behavior (12.9 h/day class 1), and less light activity (13.3 h/day class 4) compared to more light activity (12.9 h/day class 1), and more vigorous activity (13.2 h/day class 3) compared to less vigorous activity (13.1 h/day class 1).

Following development of the final LCA overall, measurement invariance was tested by group. For all sedentary behavior and physical activity classes with each of the stratification variables (age, gender, and school characteristics), the tests were significant for all solutions. This indicated that the models should be conducted by groups and, therefore, we also present stratified results.

## Average intensity

### Overall

**Fig. 1:**
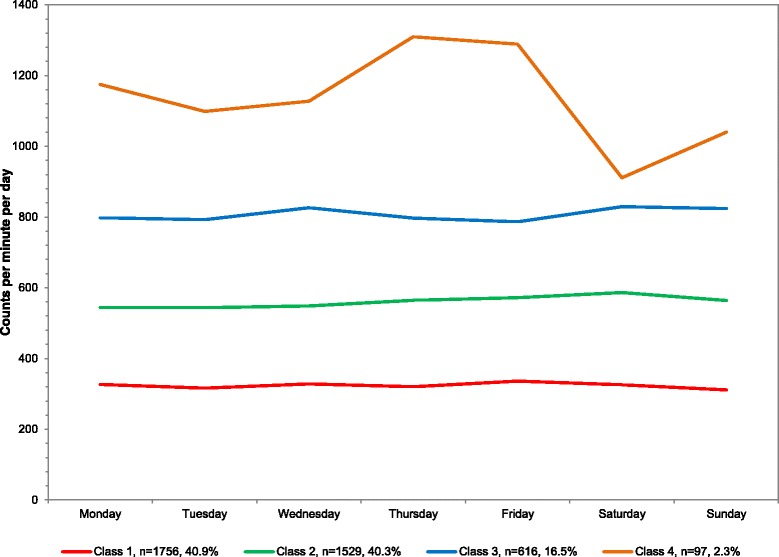
Latent class analysis plotted for weighted mean counts per minute per day among youth 6–17 years; NHANES 2003–2006

The sample averaged 507.9 counts/minute/day. For average counts/minute/day, four classes emerged from least to most active: 40.9 % of population (mean 323.5 counts/minute/day), 40.3 % (559.6), 16.5 % (810.0), and 2.3 % (1132.9) (Table 1). The average counts/minute across the week is plotted in Fig. 1 with corresponding numeric values in Additional file 1. Classes 2 to 4 were stable across all days of the week. However, the most active class (class 4) remained higher on the weekdays (1098.7 to 1309.4 average counts/minute/day) and lower on the weekends (911.0 to 1040.0 average counts/minute/day) but still higher than any other class.

**Table 1:** Descriptive information on latent classes derived from accelerometry, overall and by age, gender, and school characteristics, among youth 6–17 years; NHANES 2003-2006

	Overall		Age							Gender			In or Out of School			
				6–11 years (*n* = 1588)		12–14 years (*n* = 1247)		15–17 years (*N* = 1163		Boys(*n* = 2006)		Girls(*n* = 1992)		In School (*n* = 2874)		Out of School (*n* = 817)
	Weighted % in Class	Weighted Average CPM/Day or Min/Day	Weighted % in Class	Weighted Average CPM/Day or Min/Day	Weighted % in Class	Weighted Average CPM/Day or Min/Day	Weighted % in Class	Weighted Average CPM/Day or Min/Day	Weighted % in Class	Weighted Average CPM/Day or Min/Day	Weighted % in Class	Weighted Average CPM/Day or Min/Day	Weighted % in Class	Weighted Average CPM/Day or Min/Day	Weighted % in Class	Weighted Average CPM/Day or Min/Day
Latent class: average CPM per day																
Class 1- Least active	40.9	323.5	53.8	471.0	56.1	324.3	66.9	293.5	39.4	370.8	59.8	331.7	44.4	316.0	60.8	390.5
Class 2	40.3	559.6	41.1	746.5	35.1	554.5	26.9	516.0	40.4	605.4	37.5	624.5	37.4	532.1	36.8	720.6
Class 3	16.5	810.0	5.1	1107.4	8.8	848.3	6.2	782.7	17.7	860.4	2.7	1055.9	16.3	774.3	2.4	1124.0
Class 4 - Most active	2.3	1132.9							2.6	1147.0			1.8	1138.1		
Latent class: percent of sedentary (0- < 100 CPM) out of total wearing time per day																
Overall:																
Class 1 - Most sedentary	13.5	544.6	8.7	469.6	10.0	574.8	22.0	559.9	12.2	534.3	15.1	549.4	13.2	557.2	27.6	483.3
Class 2	30.1	455.1	35.1	391.0	37.0	478.7	48.9	486.1	33.3	441.9	29.5	466.2	29.5	476.4	49.9	363.5
Class 3	38.5	357.7	41.1	309.7	39.7	393.0	22.6	391.9	39.0	343.8	35.7	366.9	36.6	387.8	22.5	258.4
Class 4 Least sendentary	18.0	259.2	15.1	227.9	13.3	306.2	6.6	289.7	15.5	240.7	19.7	275.1	20.7	292.5		
Latent: class percent of light (100–2295) CPM) out of total wearing time per day																
Class 1 - Least light activity	12.3	222.6	14.5	298.9	31.0	248.6	38.6	228.9	10.5	221.9	13.5	222.3	16.3	228.8	29.9	253.5
Class 2	29.3	301.7	55.7	385.5	49.4	334.8	46.1	315.0	30.2	306.7	29.1	297.1	32.0	307.5	50.7	362.6
Class 3	41.8	384.0	29.8	456.3	19.6	410.6	15.3	413.0	43.4	390.1	39.1	376.2	39.1	385.4	19.4	455.9
Class 4 - Most light activity	16.6	455.5							15.9	461.4	18.3	376.2	12.6	446.2		
Latent class: percent of MVPA (> = 2296 CPM) out of total wearing time per day																
Class 1 Least 56.4MVPA	59.9	25.0	56.5	32.8	66.6	23.0	80.9	21.7	56.6	31.7	69.4	21.6	61.0	23.9	68.8	26.5
Class 2	33.3	60.9	37.8	66.6	26.4	56.4	14.2	68.2	33.8	68.4	28.7	55.7	31.4	57.8	29.0	68.1
Class 3	3.1	89.0	5.8	110.7	7.0	98.8	4.9	64.7	4.4	87.6	2.0	99.0	2.9	96.5	2.2	118.6
Class 4 - Most vigorous activity	3.6	109.3							5.1	112.0			4.7	99.0		
Latent class: of vigorous physical activity (> = 4012 CPM) out of total wearing time per day																
Class 1 Least vigorous activity	76.8	7.1	76.0	9.4	89.9	7.8	87.8	5.4	75.0	9.5	81.3	5.4	79.8	7.1	84.2	7.9
Class 2	18.5	23.9	19.7	19.7	10.1	37.0	7.7	30.4	20.0	28.0	16.3	20.4	16.7	25.5	8.8	26.7
Class 3 Most vigorous activity	4.7	47.4	4.4	49.6			4.7	38.8	5.0	50.5	2.4	44.6	3.5	50.4	7.0	37.8

*Abbreviations*: *CPM* counts per minute, *min* minute, *MVPA* moderate to vigorous physical activity

Note: In some cases, a 2-class or 3-class solution is presented rather than a 4-class solution

### Stratified

The stratified average counts/minute/day by class and day are plotted in Additional file 2 with corresponding numeric values in Additional file 1. The average counts/minute/day was lower with each successive age group (620.0 age 6–11, 468.4 age 12–14, 397.3 age 15–17), lower among girls (446.8) compared to boys (568.7), and lower among youth in school (483.1) compared to out of school (520.7). A 3-class solution emerged for each age group, with the highest average counts/minute/day class representing the smallest sample (5.1 % age 6–11, 8.8 % age 12–14, and 6.2 % age 15–17). For age 6–11, the lower two classes had consistent counts across the week, while the most active class (class 3) had more variability, ranging from 912.9 on Saturday to 1221.5 on Thursday. For age 12–14, the three classes had consistent counts across the week. For age 15–17, the lowest class (class 1) was stable across all days of the week, while the other two classes were higher on weekdays and lower on weekends.

A 4-class solution emerged for boys, but a 3-class solution fit best for girls with the most active class including only 48 girls. Most patterns were stable across the week, with the exception of the most active boys with higher average intensity on the weekdays (1115.2–1365.2 counts/minute/day) than the weekends (880.9–1016.1 counts/minute/day).

A 4-class solution emerged for in school, but a 3-class solution emerged for out of school with the most active class including only 20 youth. Both in and out of school were stable across the week for all but the most active classes, which displayed higher average intensity on the weekdays (1062.0–1269.8 in school, 1202.0–1475.7 out of school) and lower on the weekends (919.1–1138.8 in school, 646.0–760.6 out of school).

Using the final model class assignments, we compared agreement of average intensity between the overall assignment to the stratified assignment (Table 2). The weighted kappa agreement was almost perfect for all categories except for age 6–11 (fair 0.30) and out of school (moderate 0.46).

**Table 2:** Percent agreement and weighted kappa by age, gender, and school characteristics among youth 6–17 years; NHANES 2003–2006

	Age			Gender		In or Out School	
	6–11 years (*n* = 1588)	12–14 years (*n* = 1247	15–17 years (*N =* 1163)	Boys (*n* = 2006)	Girls (*n* = 1192)	In School (*n* = 2873)	Out of School (*n* = 817)
Latent class: average CPM per day							
% agreement	40.2	97.0	94.4	85.1	83.7	93.7	60.2
Weighted kappa	0.30	0.96	0.90	0.83	0.73	0.92	0.46
Latent class: percent of sedentary (0- < 100 CPM) out of total wearing time per day							
% agreement	52.2	71.0	78.9	85.2	87.7	80.6	46.5
Weighted kappa	0.48	0.67	0.75	0.85	0.88	0.80	0.39
Latent: class percent of light (100–2295) CPM) out of total wearing time per day							
% agreement	31.9	63.4	81.1	97.0	95.9	96.3	36.8
Weighted kappa	0.14	0.53	0.75	0.97	0.96	0.96	0.31
Latent class: percent of MVPA (> = 2296 CPM) out of total wearing time per day							
% agreement	86.4	93.3	88.7	88.3	94.9	94.4	86.5
Weighted kappa	0.8	0.89	0.74	0.85	0.88	0.89	0.77
Latent class: of vigorous physical activity (> = 4012 CPM) out of total wearing time per day							
% agreement	94.1	91.4	89.9	89.7	94.7	97.3	86.2
Weighted kappa	0.89	0.71	0.73	0.83	0.81	0.94	0.70

*Abbreviations*: *CPM* counts per minute

## Sedentary behavior

### Overall

**Fig. 2:**
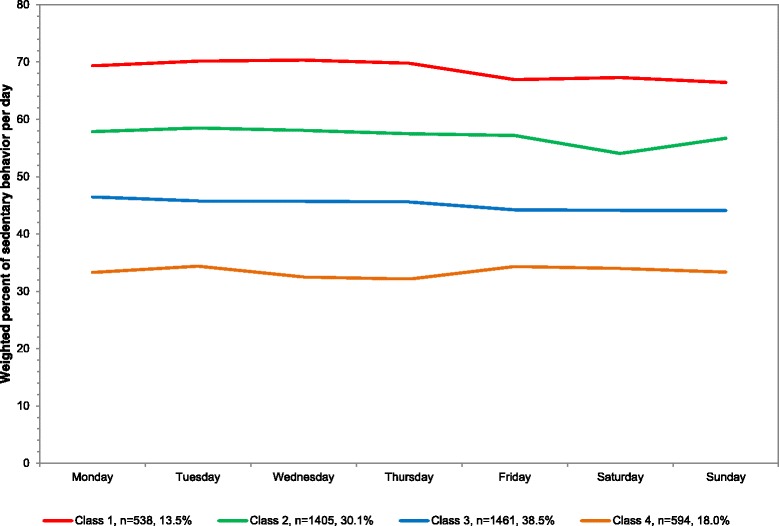
Latent class analysis plotted for weighted percent of sedentary behavior out of total wearing time per day among youth 6–17 years; NHANES 2003–2006

The sample averaged 50.9 % of sedentary behavior out of total wearing time or 6.8 h/day in sedentary behavior. For percent of sedentary behavior out of total wearing time, four classes were identified from most to least sedentary: 13.5 % of population (mean 544.6 min/day), 30.1 % (455.1), 38.5 % (357.7), and 18.0 % in the highest class (259.2) (Table 1). The mean percents across the week are plotted in Fig. 2 with corresponding numeric values in Additional file 1 and average minutes/day in Additional file 3. The percent of the day in sedentary behavior was stable across all days of the week for all four classes.

### Stratified

The percent of the day in sedentary behavior out of total wearing time was higher with each successive age group (43.2 % age 6–11, 53.9 % age 12–14, 58.1 % age 15–17), among girls (52.3 %) compared to boys (49.5 %), and when in school (52.6 %) compared to out of school (49.8 %). The stratified mean percents by class and day are plotted in Additional file 2 with corresponding numeric values in Additional file 1, along with mean minutes/day in sedentary behavior in Additional file 3.

A 4-class solution for percent time in sedentary behavior emerged for each age group. For age 6–11 years, the most sedentary class 1 had higher percent of the day in sedentary behavior on the weekdays (60.8–63.8 %) and lower on the weekends (51.9–57.5 %). Most other classes across age groups were stable across the week with one exception. For age 15–17 years, the least sedentary class 4 had a lower percent of the day in sedentary behavior on weekdays (33.7–38.5 %) and higher on weekends (40.5–50.7 %).

A 4-class solution for percent of the day in sedentary behavior emerged for boys and girls, with percent time stable across the week. A 4-class solution for percent of the day in sedentary behavior emerged for in school, but a 3-class solution emerged for out of school, with percent time stable across the week.

Using the final model class assignments, we compared agreement of sedentary behavior between the overall assignment to the stratified assignment (Table 2). Weighted kappa agreement was almost perfect for gender and in school, but lower for age (6–11 years, moderate 0.48; 12–14 years, substantial 0.67; 15–17 years, substantial 0.75) and out of school (fair 0.39).

## Light activity

### Overall

**Fig. 3:**
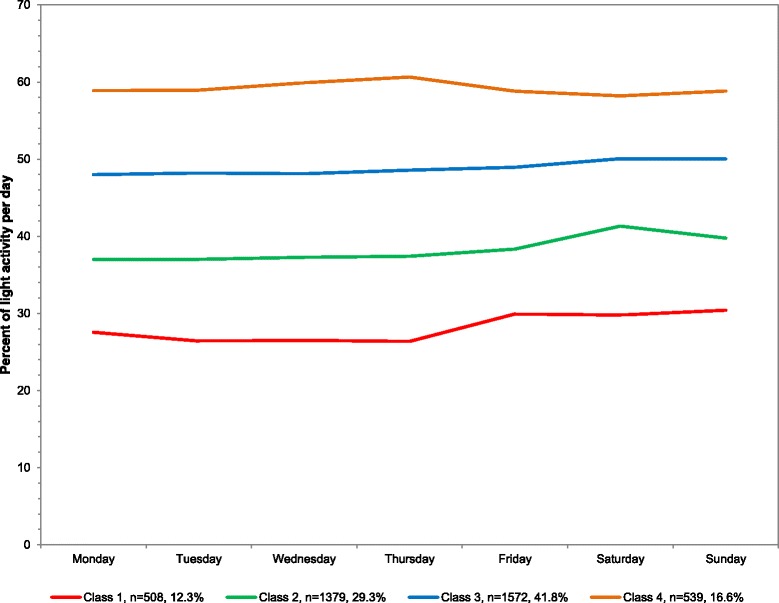
Latent class analysis plotted for weighted percent of light activity out of total wearing time per day among youth 6–17 years; NHANES 2003–2006

The sample averaged 43.8 % of light activity out of total wearing time or 349.0 min/day in light activity. For percent of light activity out of total wearing time, four classes emerged from least to most light activity: 12.3 % of population (mean 222.6 min/day), 29.3 % (301.7), 41.8 % (384.0), and 16.6 % (455.5) (Table 1). The means across the week are plotted in Fig. 3 with corresponding numeric values in Additional file 1 and average minutes/day in Additional file 3. The percent of the day in light activity was stable across all days of the week for all four classes.

### Stratified

The percent of the day in light activity out of total wearing time was lower with each successive age group (50.3 % age 6–11, 41.1 % age 12–14, 37.8 % age 15–17), but was similar by gender (43.7 % girls and 43.9 % boys) and school characteristics (42.4 % in school and 44.9 % out of school). The stratified mean percents by class and day are plotted in Additional file 2 with corresponding numeric values in Additional file 1, along with mean minutes/day in light activity in Additional file 3.

A 3-class solution emerged for each age group, with most classes stable on the weekdays and slightly higher on the weekends. However, for age 6–11 years, the most light activity class 3 was stable and high across the entire week (58.4–60.5 %) and for age 15–17 years, the most light activity class 3 was stable on the weekdays (52.1–54.7 %) and lower on the weekends (48.5–51.2 %).

A 4-class solution for percent of time in light activity emerged for boys and girls separately, with all derived classes relatively stable across the week. The light activity classes were quite similar between boys and girls. For percent of time in light activity, a 4-class solution emerged for in school and a 3-class solution emerged for out of school. For both in and out of school, the percent of time in light activity was stable across the week.

Using the final model class assignments, we compared agreement of light intensity between the overall assignment to the stratified assignment (Table 2). Weighted kappa agreement was almost perfect for gender and in school, but lower for age group (6–11 years, poor 0.14; 12–14 years, moderate 0.53; 15–17 years, substantial 0.75) and out of school (fair 0.31).

## Moderate to vigorous physical activity

### Overall

**Fig. 4:**
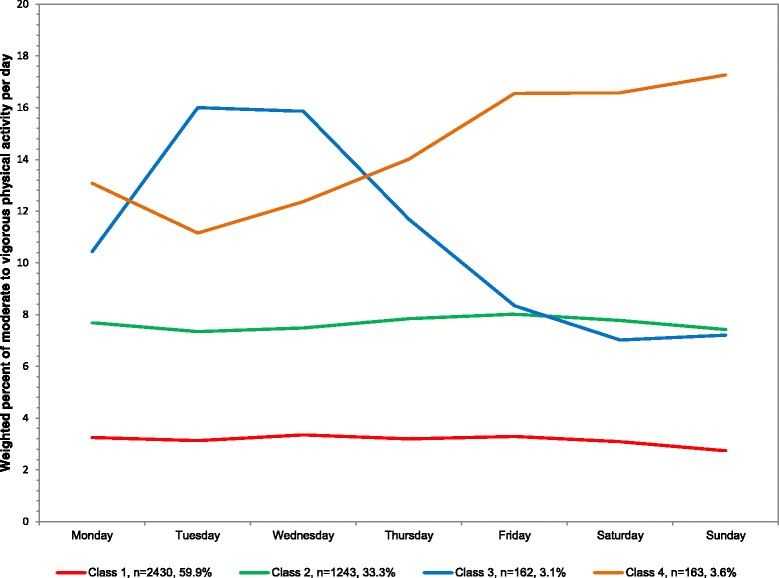
Latent class analysis plotted for weighted percent of moderate to vigorous physical activity out of total wearing time per day among youth 6–17 years; NHANES 2003–2006

The sample averaged 5.3 % of MVPA out of total wearing time or 42.7 min/day in MVPA. For percent of MVPA out of total wearing time, four classes emerged from least to most MVPA: 59.9 % of population (mean 25.0 min/day), 33.3 % (60.9), 3.1 % (89.0), and 3.6 % (109.3) (Table 1). The means across the week are plotted in Fig. 4 with corresponding numeric values in Additional file 1 and average minutes/day in Additional file 3. The percent of the day in MVPA was stable across all days of the week for the least active classes (1 and 2). Class 3 was higher on Monday through Thursday (10.4–16.0 %), but lower on Friday through Sunday (7.0–8.3 %). Class 4, the most active class, was lower on Monday through Thursday (11.2–14.0 %), but highest on Friday through Sunday (16.6–17.3 %).

### Stratified

The percent of the day in MVPA out of total wearing time was lower with each successive age group (6.5 % age 6–11, 5.0 % age 12–14, 4.1 % age 15–17) and among girls (4.1 %) compared to boys (6.6 %), but similar by school characteristics (5.1 % in school and 5.3 % out of school). A 3-class solution for percent of time in MVPA emerged for each age group. The stratified mean percents by class and day are plotted in Additional file 2 with corresponding numeric values in Additional file 1, along with mean minutes/day in MVPA in Additional file 3.

For all three age groups, the classes with the lowest MVPA were relatively stable across the week. However for the highest classes, among age 6–11 the percent time in MVPA was high Monday through Thursday (12.5–14.4 %) and even higher Friday through Sunday (15.7–16.7 %), while for age 12–14 the percent time in MVPA was high on weekdays (11.9–13.7 %) and somewhat lower on the weekend (10.3–11.4 %). For age 15–17, the middle class was relatively stable on the weekdays (7.9–10.8 %) and lower on the weekend (4.2–6.3 %), while the highest class varied across the week (4.0–15.7 %).

A 4-class solution for percent time in MVPA emerged for boys, but a 3-class solution fit best for girls with the most active class including only 41 girls. For girls, all three classes were stable across the week. For boys, the lower two classes (1 and 2) were stable across the week. However, among boys class 3 had more percent time in MVPA from Monday through Thursday (9.6–16.7 %) compared to Friday through Sunday (4.9–8.4 %), and the most active class had relatively high percent time in MVPA from Monday through Thursday (11.5–14.5 %) with even higher time on Friday through Sunday (17.0–18.0 %).

A 4-class solution for percent time in MVPA emerged for in school, but a 3-class solution emerged for out of school with the most active class including only 24 youth. For both in and out of school, the lower two classes were relatively stable across the week. For in school, class 3 was higher Monday through Thursday (10.7–16.7 %) and lower Friday through Sunday (7.0–9.0 %), while the most active class had relatively high percent time in MVPA from Monday through Thursday (9.5–11.8 %) with even higher time on Friday through Sunday (14.4–15.9 %). For a small group out of school, the most active class was high during the weekdays (14.7–17.5 %) and even higher on the weekends (18.6–21.8 %).

Using the final model class assignments, we compared agreement of MVPA between the overall assignment to the stratified assignment (Table 2). Weighted kappa agreement was almost perfect for gender, age 6–11 and 12–14 years, and in school, and slightly lower among age 15–17 (substantial 0.74) and out of school (substantial 0.77).

## Vigorous activity

### Overall

**Fig. 5:**
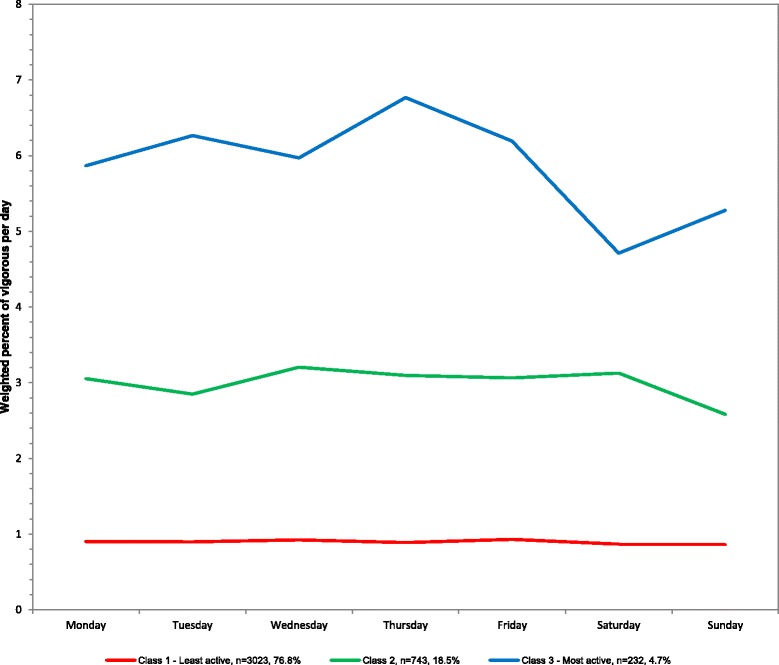
Latent class analysis plotted for weighted percent of vigorous activity out of total wearing time per day among youth 6–17 years; NHANES 2003–2006

The sample averaged 1.6 % of vigorous activity out of total wearing time or 12.7 min/day in vigorous activity. For percent of vigorous activity out of total wearing time, three classes emerged from least to most vigorous activity: 76.8 % of population (mean 7.1 min/day), 18.5 % (23.9), and 4.7 % (47.4) (Table 1). The means across the week are plotted in Fig. 5 with corresponding numeric values in Additional file 1 and average minutes/day in Additional file 3. The percent of the day in vigorous activity was stable across all days of the week for all three classes.

### Stratified

The percent of the day in vigorous activity out of total wearing time was slightly lower with each successive age group (1.9 % age 6–11, 1.5 % age 12–14, 1.2 % age 15–17), lower for girls (1.1 %) compared to boys (2.1 %), but similar by school characteristics (1.5 % in school and 1.6 % out of school). A 3-class solution emerged for each ages 6–11 and 15–17, and a 2-class solution emerged for ages 12–14, with most classes relatively stable across the week. The stratified mean percents by class and day are plotted in Additional file 2 with corresponding numeric values in Additional file 1, along with mean minutes/day in vigorous activity in Additional file 3.

A 3-class solution for percent of time in vigorous activity emerged for boys and girls separately, with the lower two classes relatively stable across the week. However, for both boys and girls, the highest classes (class 3) were lower on Saturday and Sunday. For percent of time in vigorous activity, a 3-class solution emerged for both in and out school and both were stable across the week.

Using the final model class assignments, we compared agreement of vigorous activity between the overall assignment to the stratified assignment (Table 2). Weighted kappa agreement was almost perfect for gender, ages 6–11 years, and in school, but lower for ages 12–14 years (substantial 0.71), 15–17 years (substantial 0.73), and out of school (substantial 0.70).

## Discussion

Our results indicated common ways in which physical activity and sedentary behavior can be accumulated across a week among US youth 6–17 years of age. We identified patterns for the overall sample, as well as distinct patterns by age group, gender, and whether the participant was in or out of school at the time of measurement. For average intensity, sedentary behavior, light activity, and vigorous activity, the absolute level seemed to be more important for the creation of the latent classes than the variation day-to-day. However, for MVPA, the day of week did add important information to the class assignments.

As described by others [8], the LCA approach can be viewed as a method of audience segmentation where a broad population is divided into small homogeneous groups or segments based on meaningful behavior patterns. LCA can be used to contribute to audience segmentation and provides information for framing and tailoring specific communication messages and intervention strategies. For example, in this study such messages could be tailored to the 40.9 % of youth who accumulated very little physical activity throughout the week, as indicated by low average counts/minute/day found in class 1.

Sedentary behavior, such as sitting, constitutes time spent in periods of little or no movement while awake, and at an energy expenditure ranging from 1.0 to 1.5 metabolic equivalents [14]. In the NHANES sample, sedentary behavior was higher among 15–17 year olds, girls, and those out of school. Using LCA, we identified unique groups of youth of particular concern; the most sedentary group included 13.5 % of the population and averaged 68.8 % of the day in sedentary behaviors. In the prior LCA study of adult accelerometry in NHANES, a weekend couch potato class emerged, comprised of longer bouts of sedentary behavior on the weekends, but fewer on the weekdays. The class most similar to this in the youth sample was found among 15–17 year olds, where sedentary behavior was lower on the weekdays but higher on the weekends (class 4). In a review of studies exploring physical activity, sedentary behavior, and diet contributing to obesity, Leech at al. [17] found that studies often identified a cluster defined by high levels of sedentary behavior. They suggested examining the types of sedentary behaviors that clustered with diet and physical activity by gender. For example, several studies identified a sedentary class pattern for both boys and girls, but the types of self-reported sedentary behaviors that comprised these assignments differed. In the first study, the sedentary class found for boys included more video games, computer, television, while for girls it mainly comprised computer, phone, reading [8]. In two other studies, girls were more likely to be assigned to the sedentary cluster that included socialize on the phone and have higher levels of homework, in contrast to boys who were more likely to be assigned to the high screen time cluster [18, 19].

MVPA patterns among youth were most variable across the week compared to the other four measures. Interestingly, we found some MVPA patterns were stable Monday through Thursday and then differed for Friday through Sunday. This is in contrast to US adult MVPA accelerometry patterns. For adults, if differences across the week were identified within a class, then it occurred between weekdays and weekend days, such as for the weekend warrior class [4]. In the Leech et al. [17] review, all but one study found either different clusters or classes by gender or significant differences in the proportion of boys and girls by cluster or class, with a higher proportion of boys falling into the higher clusters or classes. This finding is consistent with the accelerometer findings in our study.

## Future studies and applications

With the development of these latent class assignments, exploration into correlates and associations with other health behaviors collected in NHANES can be further explored to understand their usefulness over and above traditional time-based measures of physical activity and sedentary behavior. The patterns can be used to target interventions aimed at increasing physical activity or reducing sedentary behavior. The LCA techniques could further be applied to the NHANES data to develop (i) classes for cardiovascular- or obesity-related health behaviors that included physical activity (see review [17]), (ii) classes for physical activity, sedentary behavior, and sleep (such as Carson et al. [20]), or (iii) classes that combine self-reported and accelerometer-assessed physical activity (such as Patnode et al. [8]). In addition, LCA can be applied to accelerometry data with multiple measures to assess class patterns over time (such as Barnett et al. [21]) or on patterns within the day.

## Strengths and limitations

The strengths of the study included a national sample of youth 5–17 years with detailed measurement of physical activity and sedentary behavior. Our detailed analysis focused only on accelerometry, and provided class assignments overall and by age, gender, and school characteristics that can be used by others or applied to other data. However, this study also has several limitations. First, the uniaxial accelerometer used by NHANES under counts some activities, such as bicycling and weight lifting, and misses other activities, such as swimming, because the monitor was not waterproof and participants were told to remove it for any water-based activity. Presumably, there may be some sports that youth played in which wearing the accelerometer was prohibited.

Second, to date the most advanced cleaning protocol to remove nonwear time for the ActiGraph, in the absence of a diary indicating wear time, is an algorithm developed by Choi and colleagues among youth > =10 years and adults [11]. The research group later published a study of adults, showing that applying the algorithm using vector magnitude provided improved results than using the vertical axis only [22]. However, for the accelerometer used in NHANES only vertical counts were available. It is also unknown if the cleaning algorithm was appropriate for ages less than 10 years, although studies have applied it. Third, the cutpoints we used are only estimates of physical activity and sedentary behavior, and as mentioned in the methods section the sedentary behavior definition does include standing without moving. Newer accelerometers that provide raw data can improve upon these estimations of intensity. We extrapolated cutpoints derived on 15-second accelerometer data [12] and applied it to the 60-second data available from NHANES. Because the cutpoints were derived on steady state activities in the laboratory, the amount of error in making this translation should be minimized.

Fourth, although the overall sample size approached 4000 youth, as indicated in the results some class assignments had small sample sizes. Moreover, our attempt to explore classes by multiple groups, such as age by gender, was limited due to this issue and thus is not presented. A review paper also recognized this challenge in existing cluster analyses and recommended future studies with large enough sample sizes to overcome this challenge [17].

There are also inherent limitations to the statistical analysis that should be acknowledged. The LCA models with sampling weights applied to these data assume data are missing at random. This assumption may not always be true, for example when the accelerometer is removed for water activities. The bootstrap likelihood ratio test we used was based on unweighted data and did not account for the sampling design. However, we also used other criteria to make the final determination for the number of classes to use, including class sample size, substantive knowledge, and visual inspection.

## Conclusion

The patterns derived from this LCA provide a novel way to explore sedentary behavior and physical activity, using not only level of physical activity, but patterns across days. Many studies collect one week of accelerometry and collapse variables into weekly, weekday, or weekend measures only. This study developed latent classes that can be used by others to gain insight into the relationships between sedentary and physical activity behavior and other outcomes in the NHANES data repository or to apply to studies with similar protocols. The usefulness of this approach is that common patterns that are identified can be intervention targets by class or day of the week.

## Additional files

Additional file 1: Online Table 1: Weighted average physical activity and sedentary behavior in counts/minute or percent by day of the week for each latent class derived from accelerometry, overall and by age, gender, and school characteristics, among youth 6–17 years; NHANES 2003–2006. (PDF 56 kb) Additional file 2: Latent class analysis plotted for (1) weighted average counts/minute/day and weighted percent of (2) sedentary behavior, (3) light activity, (4) moderate to vigorous physical activity and (5) vigorous activity out of total wearing time per day, by age, gender, and school characteristics, among youth 6–17 years; NHANES 2003–2006. (PDF 144 kb) Additional file 3: Online Table 2: Weighted average physical activity and sedentary behavior in minutes/day by day of the week for each latent class derived from accelerometry, overall and by age, gender, and school characteristics, among youth 6–17 years; NHANES 2003–2006. (PDF 44 kb)

## Abbreviations

LCA: latent class analysis
MVPA: moderate to vigorous physical activity
NHANES: National Health and Nutrition Examination Survey
