# Supplementary material for: National youth sedentary behavior and physical activity daily patterns using latent class analysis applied to accelerometry
Source: Int J Behav Nutr Phys Act. 2016 May 3;13:55. doi: 10.1186/s12966-016-0382-x (PMC4855777; doi:10.1186/s12966-016-0382-x)
Supplement: Additional file 1: — Online Table 1: Weighted average physical activity and sedentary behavior in counts/minute or percent by day of the week for each latent class derived from accelerometry, overall and by age, gender, and school characteristics, among youth 6–17 years; NHANES 2003–2006. (PDF 56 kb) [file 12966_2016_382_MOESM1_ESM.pdf]

Online Table 1: Weighted average physical activity and sedentary behavior in counts/minute or percent by day of the week for each latent class derived from accelerometry, overall and by age, gender, and school characteristics, among youth 6-17 years; NHANES 2003-2006

		Weighted Average Counts/Minute or Percent							
			Day of the Week						
	n	Overall	Monday	Tuesday	Wednesday	Thursday	Friday	Saturday	Sunday
Latent class: average counts/minute/day									
Overall:									
Class 1 - Least active	1756	323.5	326.5	316.4	328.3	320.2	336.0	325.5	311.0
Class 2	1529	559.6	544.0	543.8	548.4	564.6	572.2	586.4	563.9
Class 3	616	810.0	798.1	792.7	826.4	797.1	786.5	829.3	824.0
Class 4 - Most active	97	1132.9	1174.5	1098.7	1127.6	1309.4	1288.8	911.0	1040.0
By age:									
6-11 years old									
Class 1 - Least active	863	471.0	461.8	453.8	467.7	468.8	472.2	503.0	482.0
Class 2	630	746.5	712.8	713.0	744.9	742.0	743.9	798.3	775.4
Class 3 - Most active	95	1107.4	1131.0	1100.8	1080.6	1221.5	1219.5	912.9	1086.4
12-14 years old									
Class 1 - Least active	672	324.3	331.3	319.6	327.6	323.5	335.2	320.8	305.8
Class 2	441	554.5	523.7	530.0	533.9	564.2	563.3	584.3	573.2
Class 3 - Most active	134	848.3	883.1	908.5	839.8	857.9	788.8	813.1	766.6
15-17 years old									
Class 1 - Least active	749	293.5	289.9	294.6	295.6	286.7	308.7	290.4	280.5
Class 2	329	516.0	547.0	522.7	525.2	548.6	563.6	436.4	418.8
Class 3 - Most active	85	782.7	918.4	771.0	925.4	728.6	743.5	647.0	659.6
By gender:									
Boys									
Class 1 - Least active	795	370.8	375.4	361.3	388.4	367.9	356.6	380.8	364.8
Class 2	814	605.4	584.7	581.8	602.8	608.5	628.2	641.9	588.4
Class 3	344	860.4	839.7	853.3	874.5	834.4	815.5	890.9	899.1
Class 4 - Most active	53	1147.0	1225.6	1139.3	1115.2	1275.3	1365.2	880.9	1016.1
Girls									
Class 1 - Least active	1301	331.7	332.5	329.2	328.2	330.7	353.2	326.3	318.7
Class 2	643	624.5	607.5	604.1	597.9	634.9	642.3	654.3	642.6
Class 3 - Most active	48	1055.9	1062.4	998.5	1156.1	1132.9	1001.4	946.1	1060.5
By school characteristics:									
In school									
Class 1 - Least active	1321	316.0	313.8	309.4	320.3	316.3	328.4	320.4	305.3
Class 2	1058	532.1	532.1	526.9	522.8	544.0	544.7	532.6	519.5
Class 3	437	774.3	716.4	719.2	780.9	787.5	767.1	848.0	807.3
Class 4 - Most active	58	1138.1	1200.0	1140.4	1062.0	1269.8	1224.3	919.1	1138.8
Out school									

Class 1 - Least active	514	390.5	403.1	391.8	396.0	383.1	400.4	391.2	371.0
Class 2	283	720.6	691.1	721.2	736.8	698.1	713.2	738.1	715.1
Class 3 - Most active	20	1124.0	1223.6	1204.3	1289.2	1475.7	1202.0	760.6	646.0
Latent class: percent of sedentary (0-<100 counts/minute) out of total wearing time per day									
Overall:									
Class 1 - Most sedentary	538	68.8	69.3	70.2	70.4	69.8	66.9	67.3	66.4
Class 2	1405	57.3	57.9	58.5	58.1	57.5	57.2	54.1	56.7
Class 3	1461	45.2	46.5	45.8	45.7	45.6	44.2	44.1	44.1
Class 4 -Least sedentary	594	33.3	33.3	34.4	32.5	32.1	34.3	34.0	33.3
By age:									
Age 6-11 years old									
Class 1 - Most sedentary	120	60.5	61.3	63.8	61.9	60.8	61.9	51.9	57.5
Class 2	587	49.4	50.1	49.8	50.1	50.3	48.0	48.3	48.3
Class 3	640	39.3	40.6	40.4	39.7	38.8	40.2	37.6	37.1
Class 4 -Least sedentary	241	29.6	29.3	30.5	28.6	28.6	30.3	30.6	30.4
Age 12-14 years old									
Class 1 - Most sedentary	103	71.3	72.2	71.6	71.9	72.1	68.8	68.7	70.2
Class 2	463	60.5	60.9	62.3	62.7	61.2	59.0	57.6	58.1
Class 3	518	49.8	50.7	50.7	49.8	49.2	50.0	48.3	49.9
Class 4 -Least sedentary	163	39.1	42.7	38.2	38.8	37.5	36.9	38.7	40.9
Age 15-17 years old									
Class 1 - Most sedentary	204	70.9	71.6	72.8	73.1	73.3	68.0	69.4	67.4
Class 2	605	60.7	61.4	61.2	60.1	61.0	60.6	59.0	60.8
Class 3	290	49.0	49.6	49.2	49.3	48.1	47.2	47.2	52.2
Class 4 -Least sedentary	64	37.7	33.7	38.5	35.5	36.8	36.4	50.7	40.5
By gender:									
Boys									
Class 1 - Most sedentary	267	67.9	68.7	69.0	69.0	67.7	66.3	67.2	66.2
Class 2	743	55.2	55.8	55.8	56.0	55.8	55.5	51.5	54.5
Class 3	741	43.1	44.0	43.3	42.7	43.1	43.0	42.7	42.6
Class 4 -Least sedentary	255	31.0	31.0	31.3	30.8	30.2	31.4	31.8	32.1
Girls									
Class 1 - Most sedentary	275	69.3	69.4	70.9	71.4	71.0	67.5	66.7	66.7
Class 2	693	58.8	59.4	60.3	59.9	58.7	57.9	56.5	57.7
Class 3	697	47.0	48.5	48.0	47.8	47.3	45.5	45.9	45.5
Class 4 -Least sedentary	327	35.3	35.9	37.0	34.6	34.6	36.2	34.8	34.7
By school characteristics:									
In school									
Class 1 - Most sedentary	341	69.7	70.0	70.5	72.1	70.6	67.9	68.3	66.8
Class 2	978	59.3	60.1	61.0	59.9	59.6	58.6	55.7	58.2
Class 3	1058	48.2	49.2	49.2	48.6	48.3	47.6	46.9	47.2
Class 4 -Least sedentary	497	37.1	38.1	38.5	36.6	36.3	37.2	36.5	36.4
Out school									

Class 1 - Most sedentary	239	63.7	63.7	64.4	63.6	64.9	62.9	61.8	64.0
Class 2	415	47.8	48.3	47.4	48.7	48.0	47.5	46.3	48.8
Class 3 -Least sedentary	163	33.4	34.0	34.9	32.4	32.6	33.8	33.5	33.9
Latent class: percent of light (100-2295 counts/minute) out of total wearing time per day									
Overall:									
Class 1 - Least light activity	508	27.9	27.6	26.4	26.5	26.4	29.9	29.8	30.4
Class 2	1379	38.1	37.0	37.0	37.3	37.4	38.3	41.3	39.8
Class 3	1572	48.8	48.0	48.2	48.1	48.6	49.0	50.1	50.1
Class 4 - Most light activity	539	59.2	58.9	58.9	59.9	60.6	58.8	58.2	58.8
By age:									
Age 6-11 years old									
Class 1 - Least light activity	228	37.9	37.5	35.9	36.8	37.1	37.6	43.3	40.4
Class 2	906	49.0	48.1	48.7	48.2	48.2	49.0	50.1	50.8
Class 3 - Most light activity	454	59.2	58.9	58.4	59.6	60.5	58.7	59.1	59.2
Age 12-14 years old									
Class 1 - Least light activity	363	31.4	30.4	30.0	29.5	30.8	32.7	33.4	34.9
Class 2	662	42.3	41.5	40.8	41.5	41.8	42.5	45.3	42.9
Class 3 - Most light activity	222	52.5	50.1	52.7	52.8	53.7	54.4	52.6	52.1
Age 15-17 years old									
Class 1 - Least light activity	409	28.8	28.4	26.8	26.6	27.2	30.5	31.5	31.4
Class 2	586	39.6	38.3	39.5	39.5	39.1	39.7	41.5	39.4
Class 3 - Most light activity	168	52.8	52.6	52.1	54.4	54.7	53.9	48.5	51.2
By gender:									
Boys									
Class 1 - Least light activity	247	27.8	27.1	26.3	26.9	26.7	29.9	29.3	29.8
Class 2	706	38.4	37.4	37.9	37.8	37.7	38.2	41.8	40.0
Class 3	808	49.1	48.6	48.9	48.4	48.9	48.8	49.8	50.1
Class 4 - Most light activity	245	59.9	59.7	60.4	60.5	61.6	59.7	58.4	58.7
Girls									
Class 1 - Least light activity	252	27.8	27.6	26.1	25.8	26.2	29.7	30.3	31.0
Class 2	678	37.6	36.7	35.7	36.5	37.1	38.5	40.4	39.5
Class 3	759	48.4	47.1	47.1	47.7	47.9	49.1	50.2	50.1
Class 4 - Most light activity	303	48.4	57.6	57.7	58.7	59.4	57.6	58.1	58.2
By school characteristics:									
In school									
Class 1 - Least light activity	468	28.4	27.9	27.5	26.4	27.1	29.9	30.4	31.4
Class 2	1061	38.3	37.4	36.5	37.6	37.7	38.8	41.9	40.3
Class 3	1042	48.2	46.9	47.2	47.6	47.9	48.5	50.1	50.0
Class 4 - Most light activity	303	57.7	57.9	56.9	58.7	59.0	57.3	56.0	57.2
Out school									
Class 1 - Least light activity	266	33.5	32.5	33.3	32.9	32.6	33.6	36.3	34.1
Class 2	414	47.7	47.9	47.9	47.3	48.0	47.8	48.4	46.8
Class 3 - Most light activity	137	59.5	58.9	57.8	60.2	60.6	59.6	60.1	59.4

Latent class: percent of moderate to vigorous physical activity (>=2296 counts/minute) out of total wearing time per day									
Overall:									
Class 1 - Least active	2430	3.2	3.3	3.1	3.4	3.2	3.3	3.1	2.7
Class 2	1243	7.7	7.7	7.3	7.5	7.8	8.0	7.8	7.4
Class 3	162	11.4	10.4	16.0	15.9	11.7	8.3	7.0	7.2
Class 4 - Most active	163	14.2	13.1	11.2	12.4	14.0	16.6	16.6	17.3
By age:									
Age 6-11 years old									
Class 1 - Least active	886	4.2	4.2	4.1	4.2	4.2	4.3	4.4	4.0
Class 2	586	8.5	8.2	8.1	8.7	8.7	8.6	8.8	8.4
Class 3 - Most active	116	14.4	12.9	12.5	14.4	13.8	15.7	15.9	16.7
Age 12-14 years old									
Class 1 - Least active	783	2.9	3.0	2.9	3.1	3.0	3.1	2.6	2.3
Class 2	348	7.0	6.7	7.3	6.6	7.1	7.0	7.0	7.0
Class 3 - Most active	116	12.4	13.7	11.9	12.7	13.6	12.2	11.4	10.3
Age 15-17 years old									
Class 1 - Least active	903	2.7	2.9	2.8	3.0	2.7	3.0	2.3	1.9
Class 2	195	8.5	10.8	7.9	9.7	9.6	8.6	6.3	4.2
Class 3 - Most active	65	8.5	4.9	11.5	7.5	7.2	8.3	4.0	15.7
By gender:									
Boys									
Class 1 - Least active	1091	4.0	4.1	3.7	4.4	4.0	4.0	4.0	3.4
Class 2	679	8.6	8.6	8.0	8.4	8.5	8.8	8.8	8.6
Class 3	123	11.1	9.6	16.7	15.6	12.4	8.4	6.4	4.9
Class 4 - Most active	113	14.6	12.9	11.5	12.7	14.5	18.0	18.0	17.0
Girls									
Class 1 - Least active	1437	2.7	2.8	2.9	2.8	2.8	3.1	2.5	2.3
Class 2	514	7.1	7.1	6.7	6.9	7.6	7.5	7.0	6.8
Class 3 - Most active	41	13.1	13.9	14.9	14.6	10.8	9.3	10.4	17.3
By school characteristics:									
In school									
Class 1 - Least active	1739	3.0	3.1	3.0	3.2	3.1	3.1	2.7	2.4
Class 2	892	7.2	7.1	6.9	7.1	7.7	7.6	7.1	6.6
Class 3	108	12.0	10.7	15.9	16.7	12.9	9.0	7.0	8.0
Class 4 - Most active	135	12.6	11.8	9.5	11.0	11.7	14.4	15.9	15.7
Out school									
Class 1 - Least active	559	3.5	3.5	3.4	3.5	3.5	3.9	3.8	3.0
Class 2	234	8.7	8.8	9.3	9.3	8.3	8.5	7.9	8.3
Class 3 - Most active	24	16.5	17.5	15.9	15.5	14.7	15.5	21.8	18.6
Latent class: percent of vigorous physical activity (>=4012 counts/minute) out of total wearing time per day									
Overall:									
Class 1 - Least active	3023	0.9	0.9	0.9	0.9	0.9	0.9	0.9	0.9

Class 2	743	3.0	3.1	2.9	3.2	3.1	3.1	3.1	2.6
Class 3 - Most active	232	6.0	5.9	6.3	6.0	6.8	6.2	4.7	5.3
By age:									
Age 6-11 years old									
Class 1 - Least active	1187	1.2	1.2	1.1	1.2	1.2	1.2	1.2	1.2
Class 2	314	3.3	2.8	3.1	3.4	3.5	3.4	3.7	2.9
Class 3 - Most active	87	6.2	6.2	5.9	6.6	7.1	6.4	5.0	6.3
Age 12-14 years old									
Class 1 - Less active	1082	1.0	0.9	1.0	1.0	1.0	1.0	0.8	0.9
Class 2 - More active	165	4.6	4.7	5.0	4.4	4.6	4.3	5.0	3.9
Age 15-17 years old									
Class 1 - Least active	992	0.7	0.7	0.7	0.8	0.7	0.7	0.4	0.5
Class 2	104	3.8	6.4	3.9	5.0	3.7	3.6	1.1	2.0
Class 3 - Most active	67	4.8	3.5	4.5	4.1	5.2	4.8	6.8	4.0
By gender:									
Boys									
Class 1 - Least active	1408	1.2	1.2	1.2	1.4	1.2	1.2	1.1	1.1
Class 2	464	3.5	3.5	3.5	3.7	3.4	3.6	3.8	3.0
Class 3 - Most active	134	6.3	6.0	6.0	6.7	8.2	6.7	5.0	5.2
Girls									
Class 1 - Least active	1674	0.7	0.7	0.7	0.6	0.7	0.7	0.7	0.7
Class 2	272	2.6	2.7	2.3	2.5	3.1	2.7	2.7	2.2
Class 3 - Most active	46	5.7	7.3	7.1	5.7	5.6	4.7	3.5	5.9
By school characteristics:									
In school									
Class 1 - Least active	2263	0.9	0.9	0.9	0.9	0.9	0.9	0.8	0.8
Class 2	482	3.2	3.0	2.8	3.4	3.3	3.4	3.5	2.8
Class 3 - Most active	129	6.2	5.6	7.1	5.5	8.0	6.4	5.0	4.9
Out school									
Class 1 - Least active	671	1.0	0.9	1.0	1.1	1.0	1.1	1.1	1.0
Class 2	83	3.5	6.8	3.0	3.7	2.0	3.3	1.8	3.5
Class 3 - Most active	63	4.9	3.5	6.7	5.1	6.3	4.0	4.9	3.0
