# Supplementary material for: National youth sedentary behavior and physical activity daily patterns using latent class analysis applied to accelerometry
Source: Int J Behav Nutr Phys Act. 2016 May 3;13:55. doi: 10.1186/s12966-016-0382-x (PMC4855777; doi:10.1186/s12966-016-0382-x)

Title of Paper: Youth Physical Activity and Sedentary Behavior Weekly Patterns Using Accelerometry from a National Sample

Additional File Figures:

Online Figures 1a, b, c: Latent class analysis plotted for weighted average counts per minute per day; NHANES 2003-2006

- Page 2 1a: by age group
- Page 3 1b: by gender
- Page 4 1c: by in or out of school

Online Figures 2a, b, c: Latent class analysis plotted for weighted percent of sedentary activity out of total wearing time per day; NHANES 2003-2006

- Page 5 2a: by age group
- Page 6 2b: by gender
- Page 7 2c: by in or out of school

Online Figures 3a, b, c: Latent class analysis plotted for weighted percent of light activity out of total wearing time per day; NHANES 2003-2006

- Page 8 3a: by age group
- Page 9 3b: by gender
- Page 10 3c: by in or out of school

Online Figures 4a, b, c: Latent class analysis plotted for weighted percent of moderate to vigorous physical activity out of total wearing time per day; NHANES 2003-2006

- Page 11 4a: by age group
- Page 12 4b: by gender
- Page 13 4c: by in or out of school

Online Figures 5a, b, c: Latent class analysis plotted for weighted percent of vigorous physical activity out of total wearing time per day; NHANES 2003-2006

- Page 14 5a: by age group
- Page 15 5b: by gender
- Page 16 5c: by in or out of school

Online Figure 1a: Latent class analysis plotted for weighted average counts per minute per day by age groups; NHANES 2003-2006

Latent class pattern	Weighted %

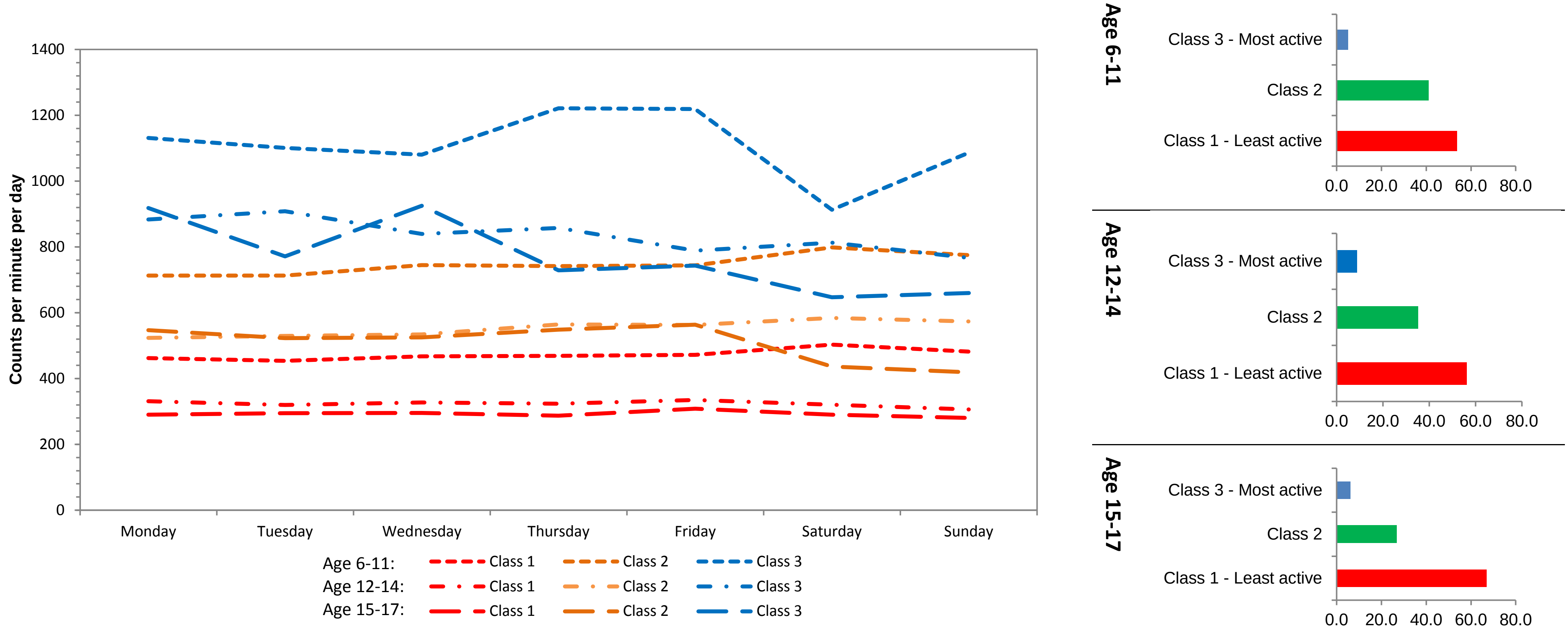

Online Figure 1b: Latent class analysis plotted for weighted average counts per minute per day by gender; NHANES 2003-2006

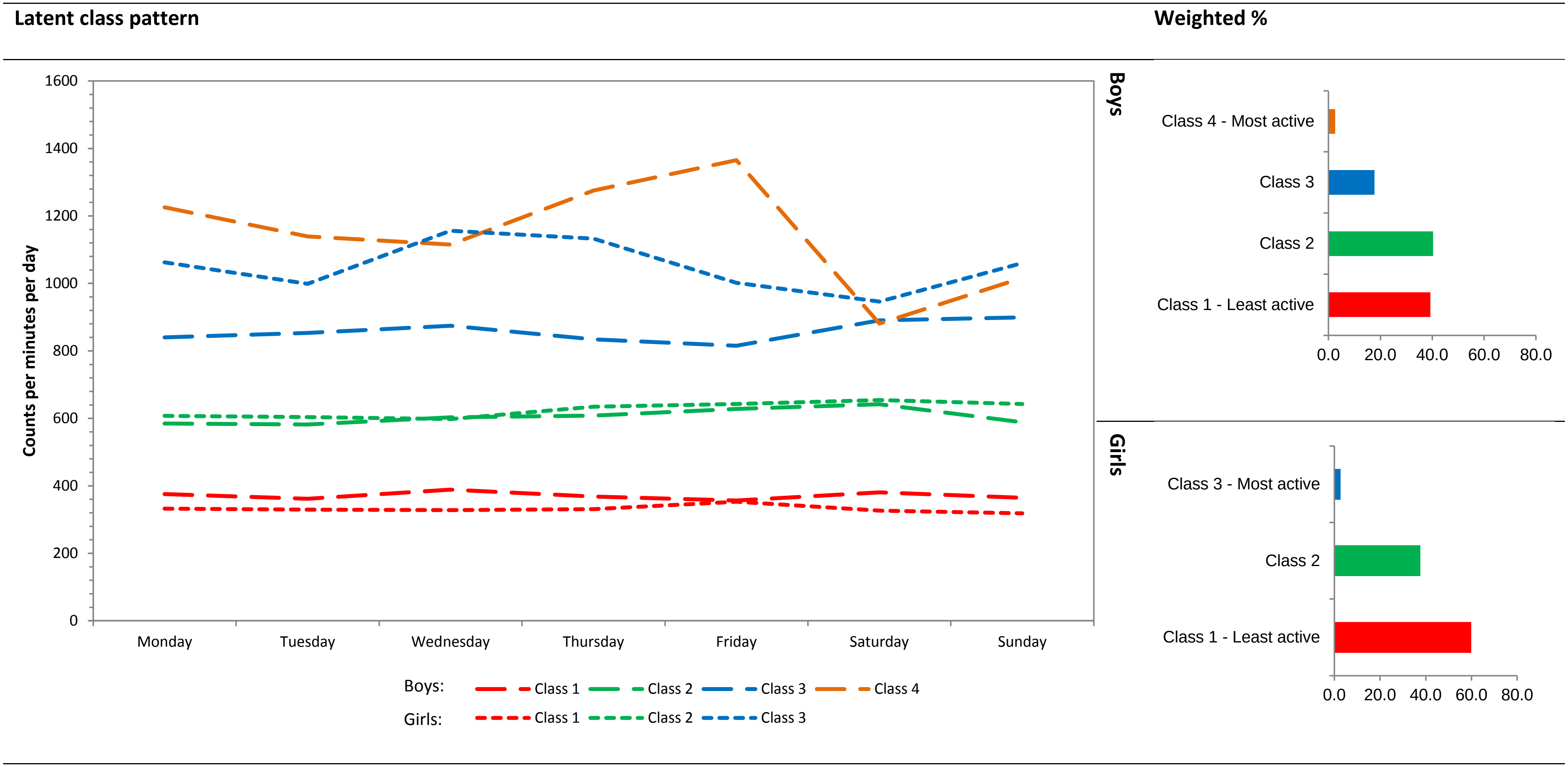

Online Figure 1c: Latent class analysis plotted for weighted mean counts per minute per day by in or out of school; NHANES 2003-2006

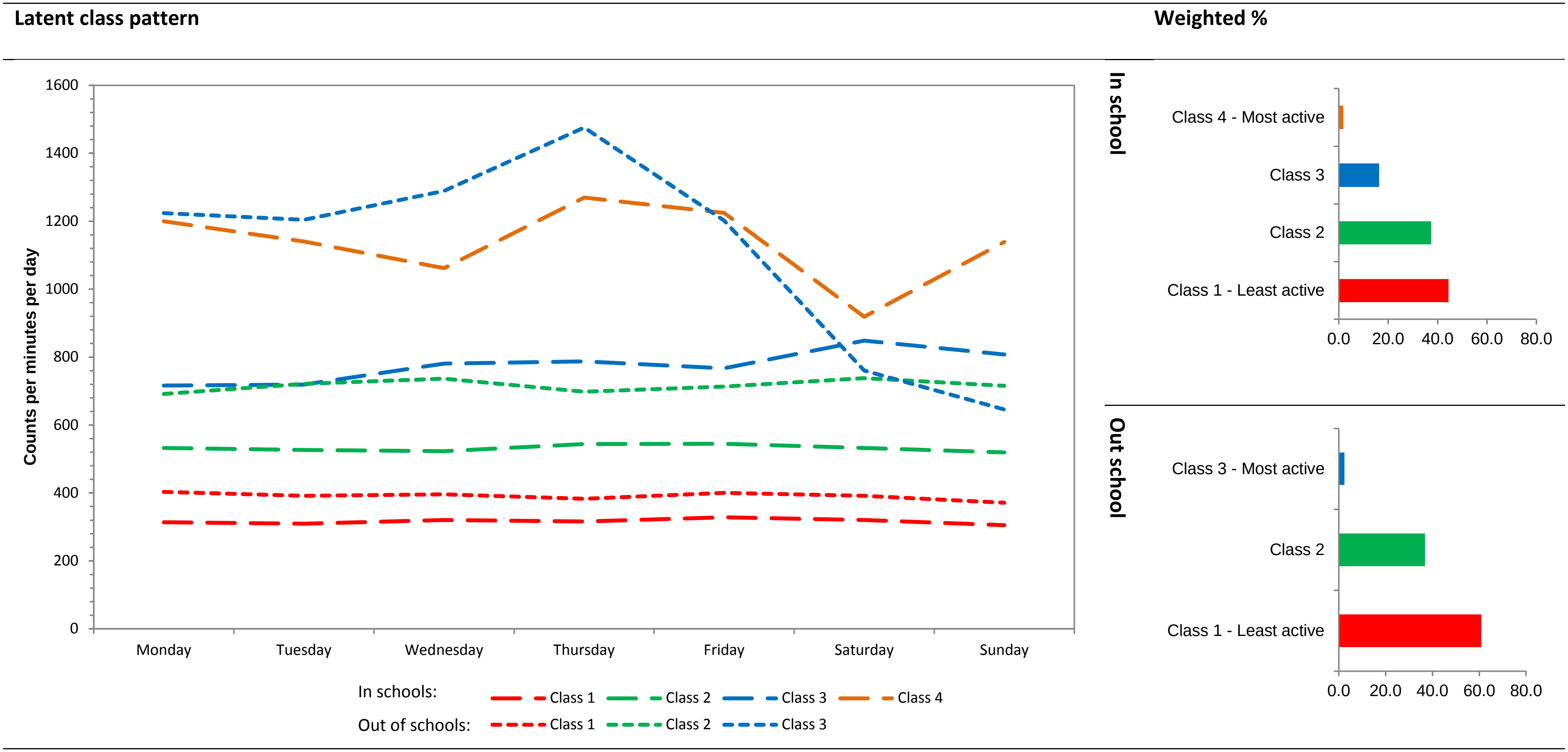

Figure 2a: Latent class analysis plotted for weighted percent of sedentary behavior out of total wearing time per day by age groups; NHANES 2003-2006

Latent class pattern **Weighted %**

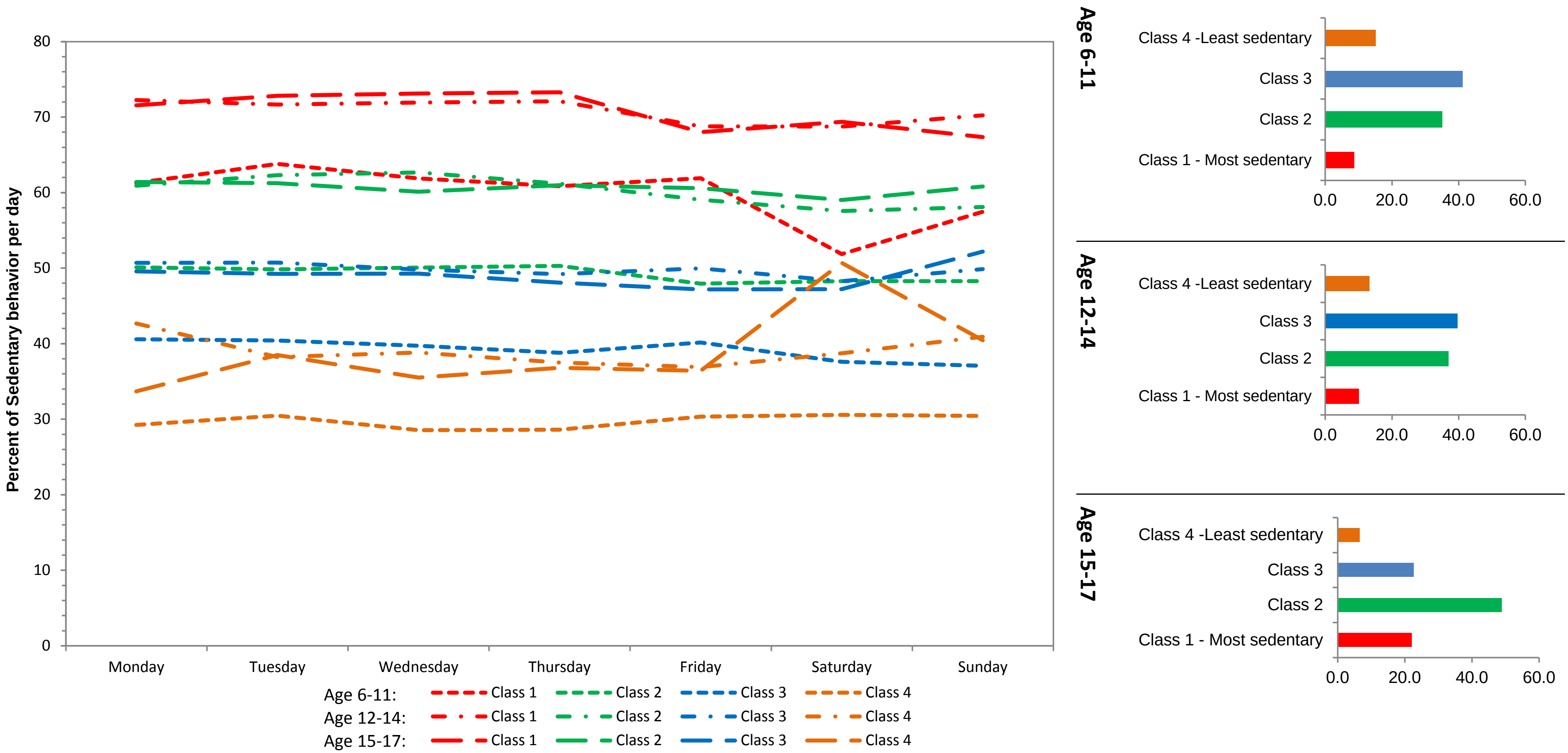

Online Figure 2b: Latent class analysis plotted for weighted percent of sedentary behavior out of total wearing time per day by gender; NHANES 2003-2006

**Latent class pattern**	**Weighted %**

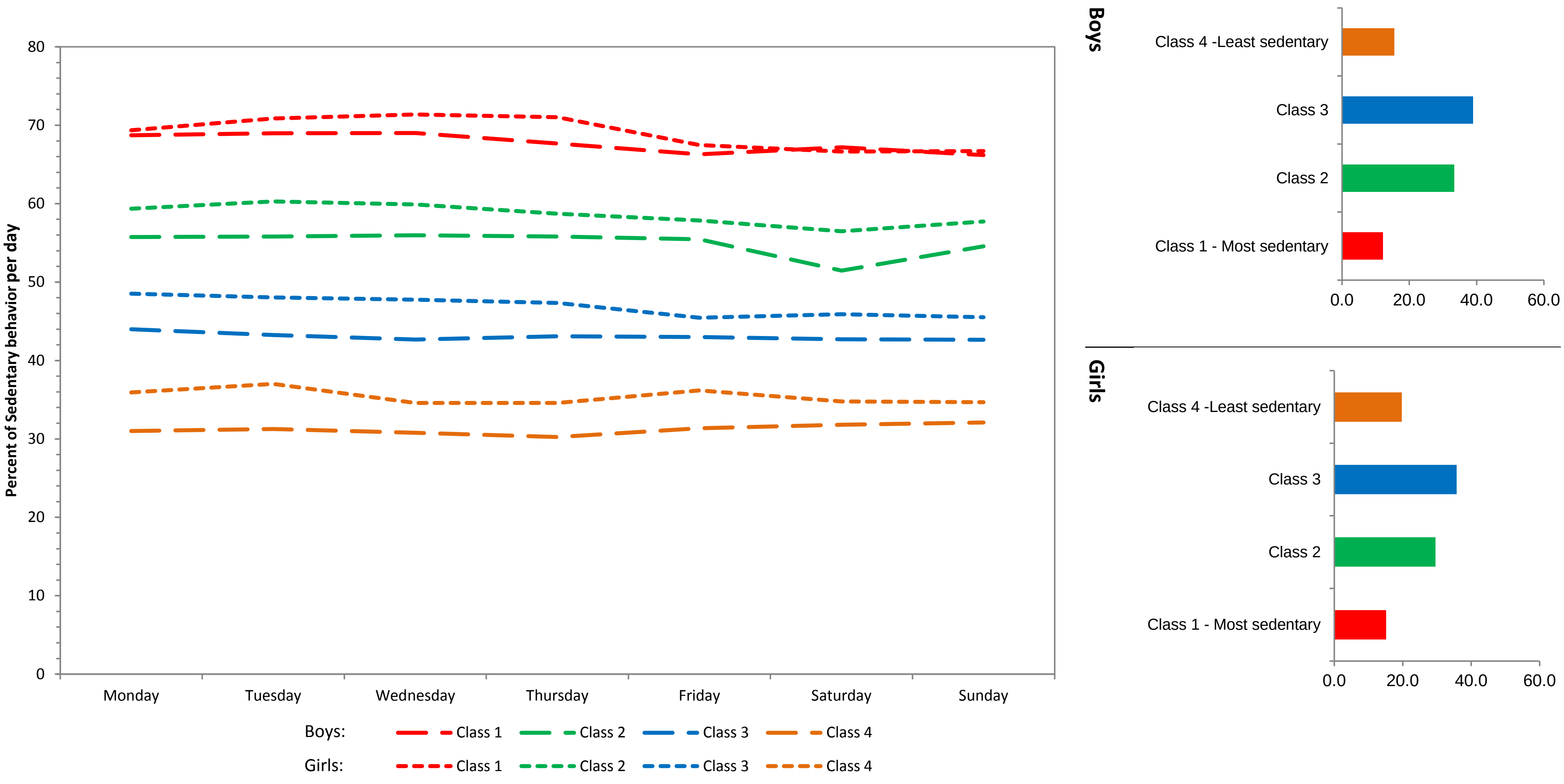

Online Figure 2c: Latent class analysis plotted for weighted percent of sedentary behavior out of total wearing time per day by in or out of school; NHANES 2003-2006

**Latent class pattern**	**Weighted %**

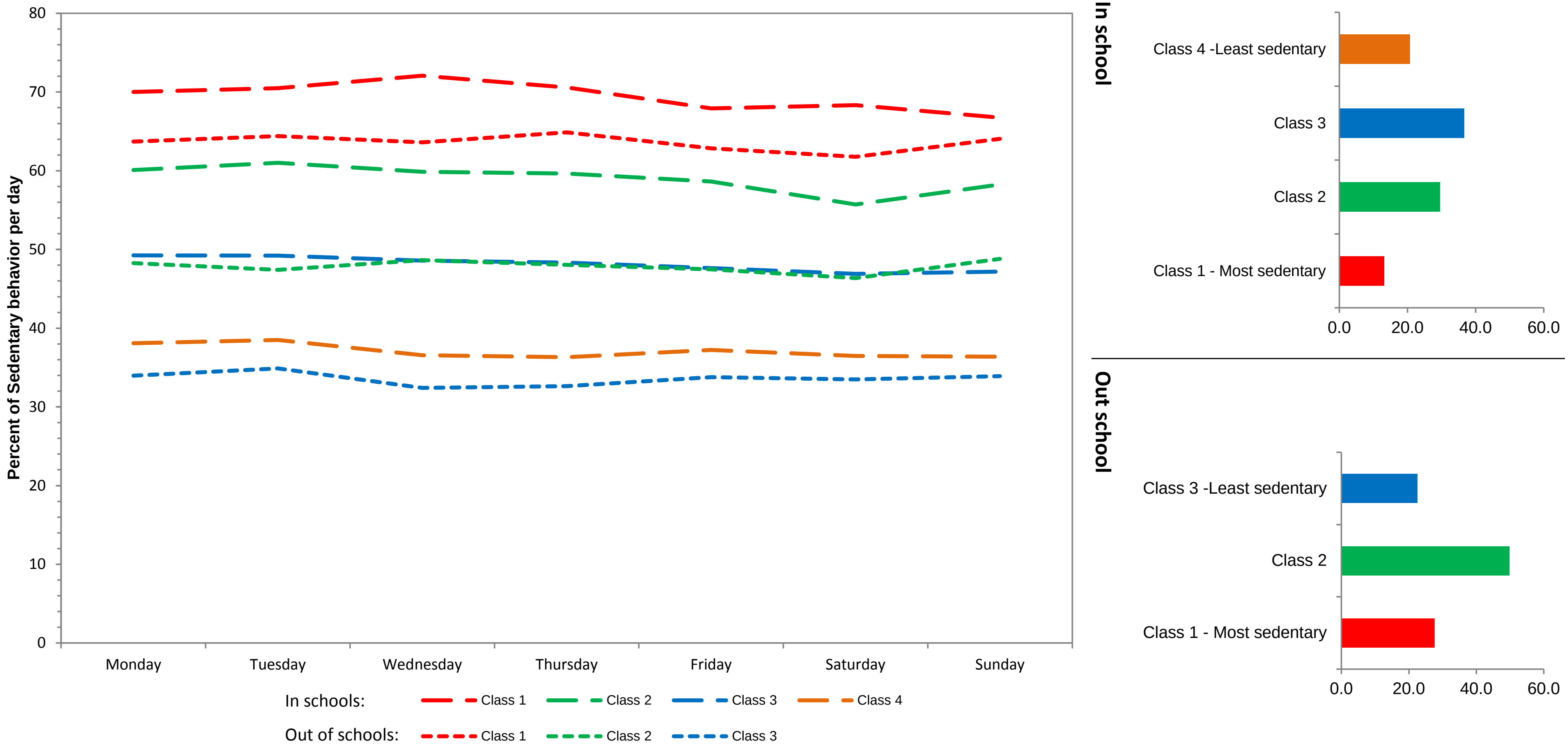

Online Figure 3a: Latent class analysis plotted for weighted percent of light activity out of total wearing time per day by age groups; NHANES 2003-2006

Latent class pattern	Weighted %

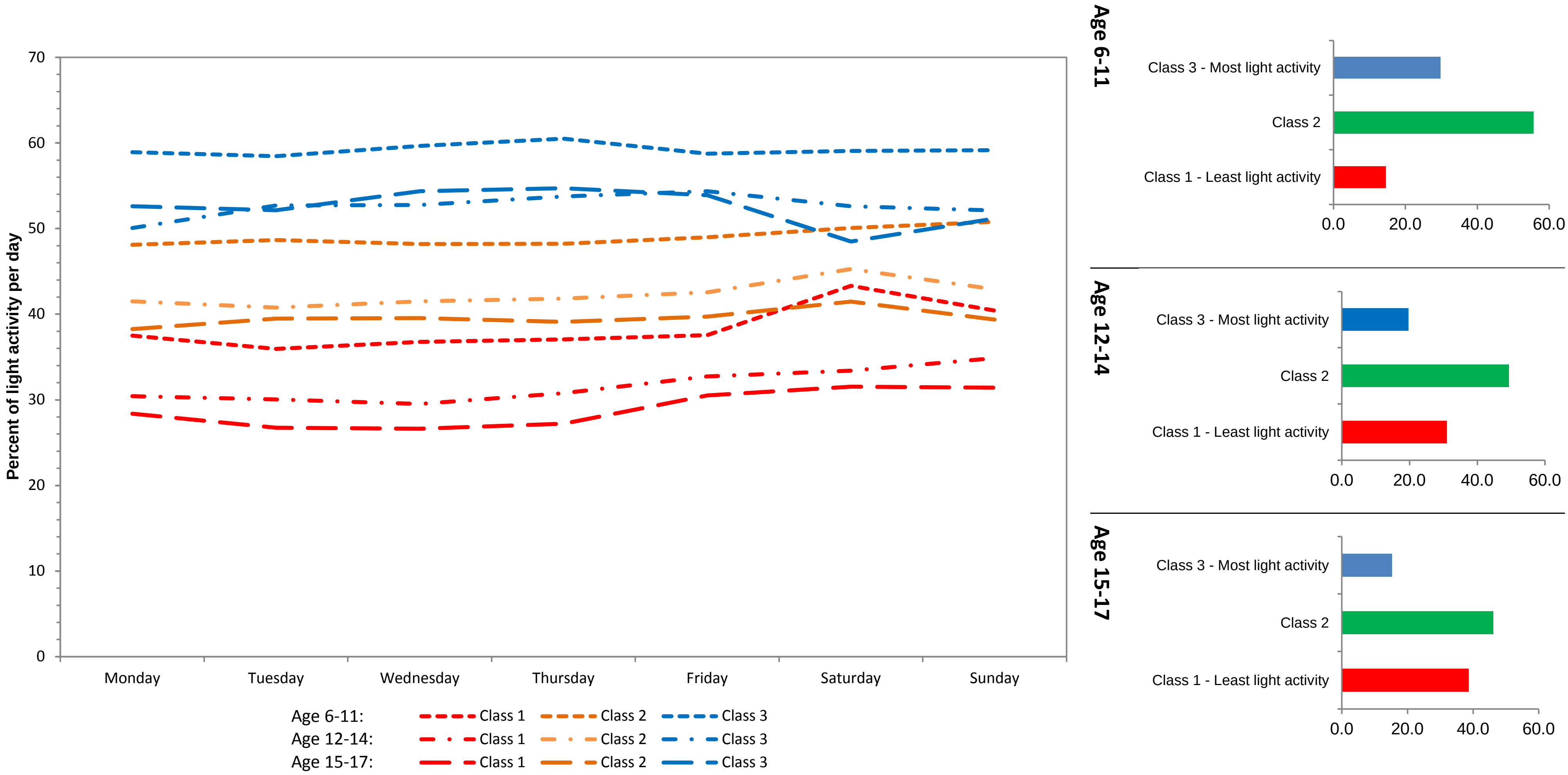

Online Figure 3b: Latent class analysis plotted for weighted percent of light activity out of total wearing time per day by gender; NHANES 2003-2006

Latent class pattern	Weighted %

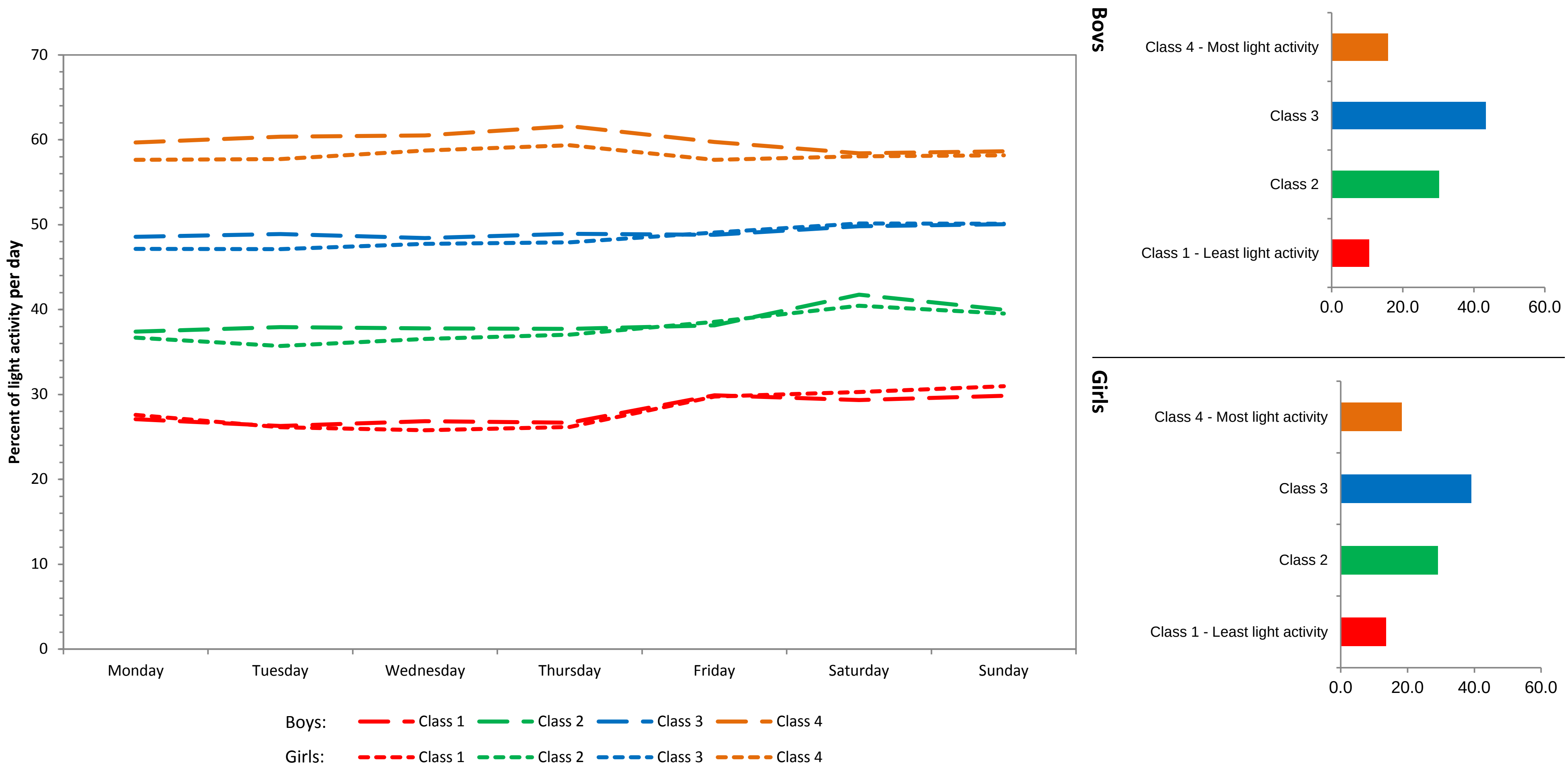

Online Figure 3c: Latent class analysis plotted for weighted percent of light activity out of total wearing time per day by in or out of school; NHANES 2003-2006

Latent class pattern **Weighted %**

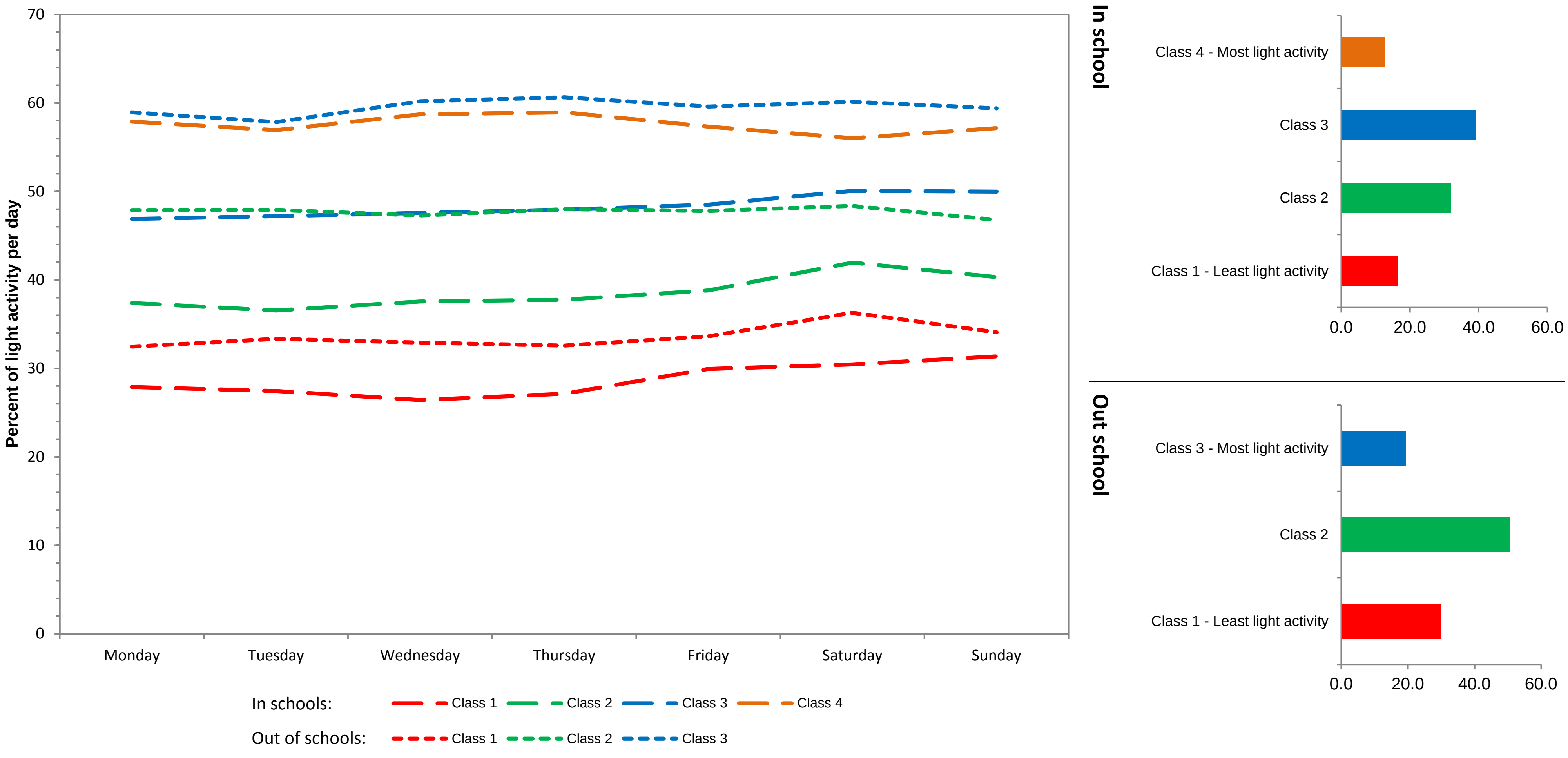

Online Figure 4a: Latent class analysis plotted for weighted percent of moderate to vigorous physical activity out of total wearing time per day by age groups; NHANES 2003-2006

Latent class pattern | **Weighted %**

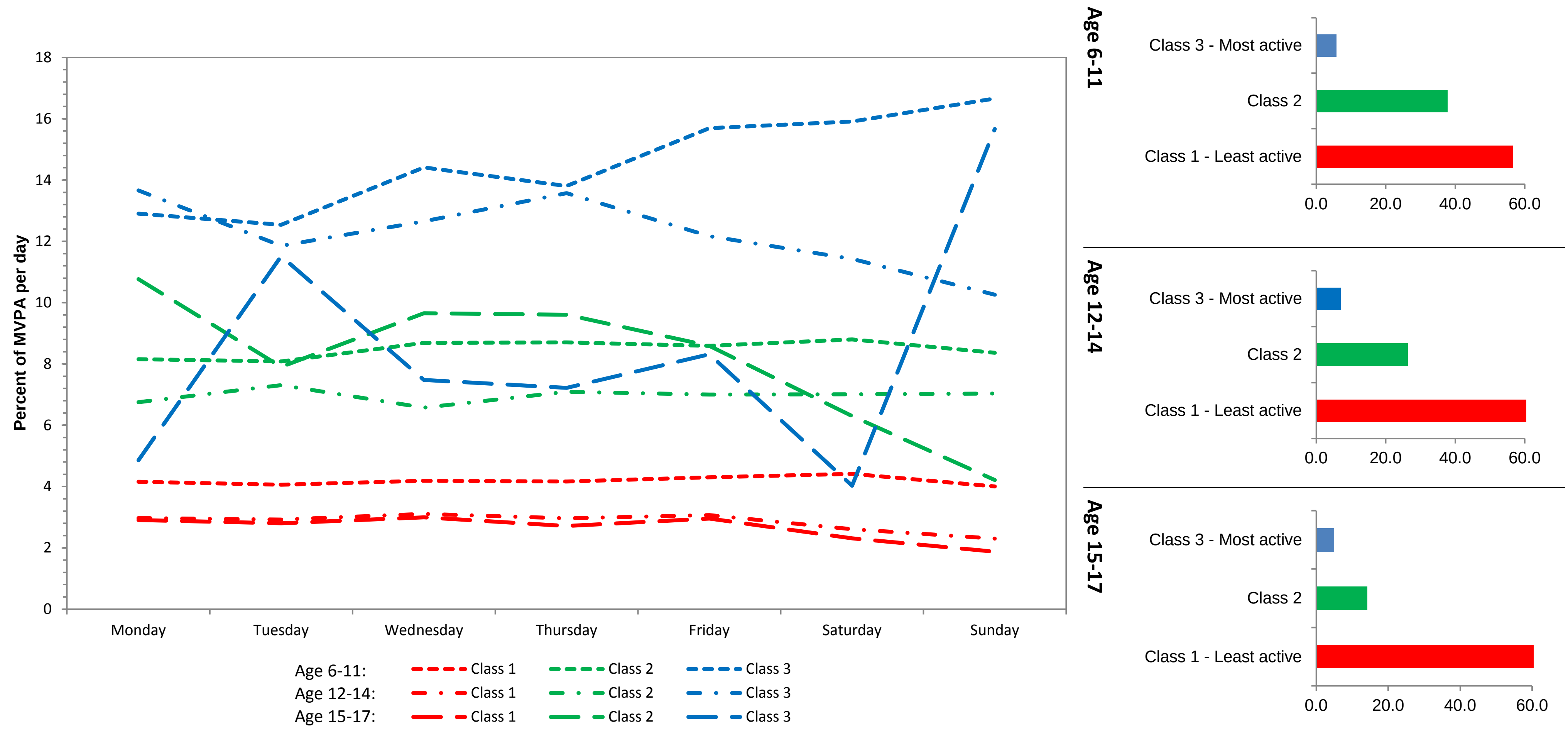

Online Figure 4b: Latent class analysis plotted for weighted percent of moderate to vigorous physical activity out of total wearing time per day by gender; NHANES 2003-2006

Latent class pattern | **Weighted %**

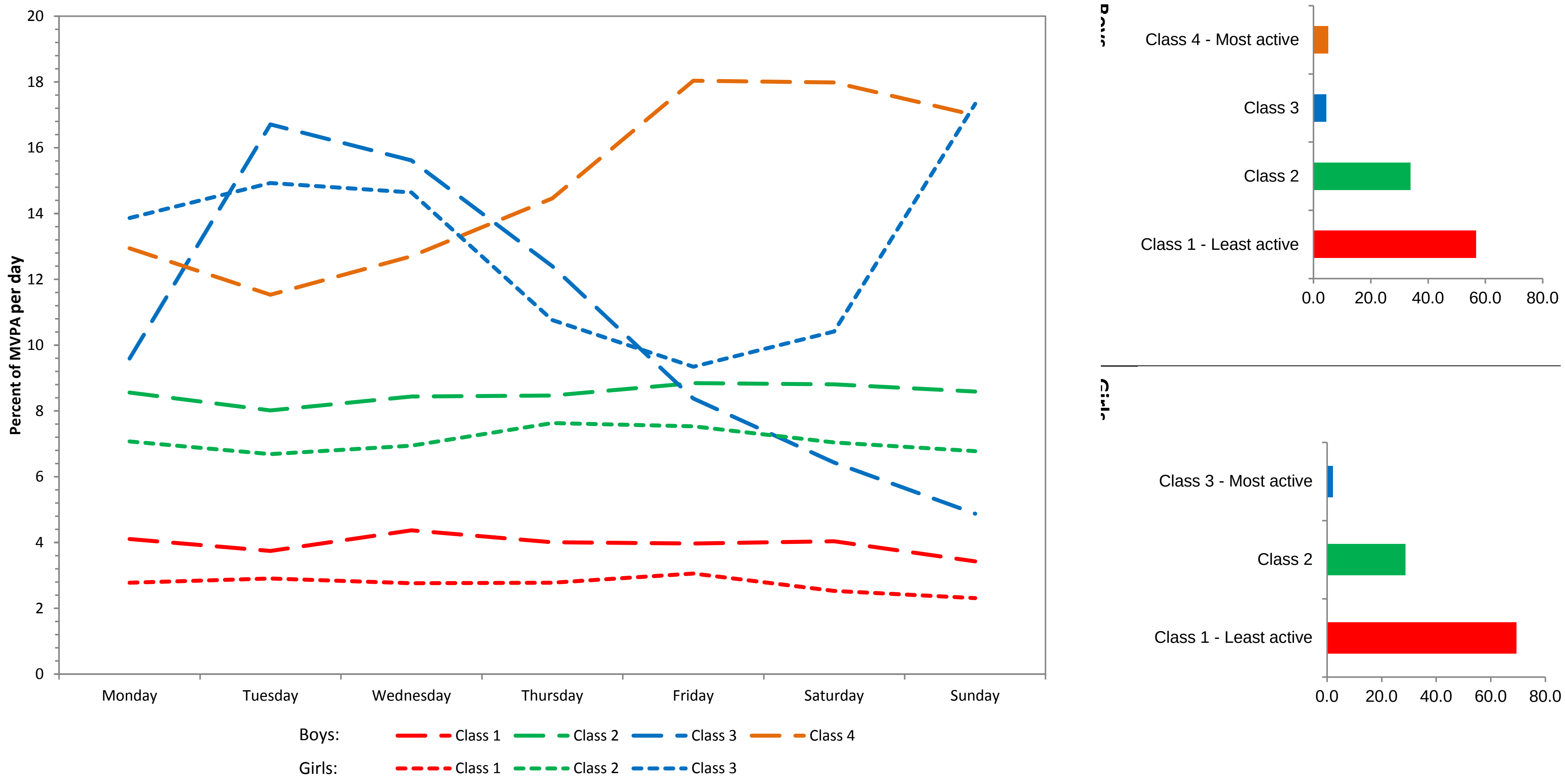

Online Figure 4c: Latent class analysis plotted for weighted percent of moderate to vigorous physical activity out of total wearing time per day by in or out of school; NHANES 2003-2006

Latent class pattern **Weighted %**

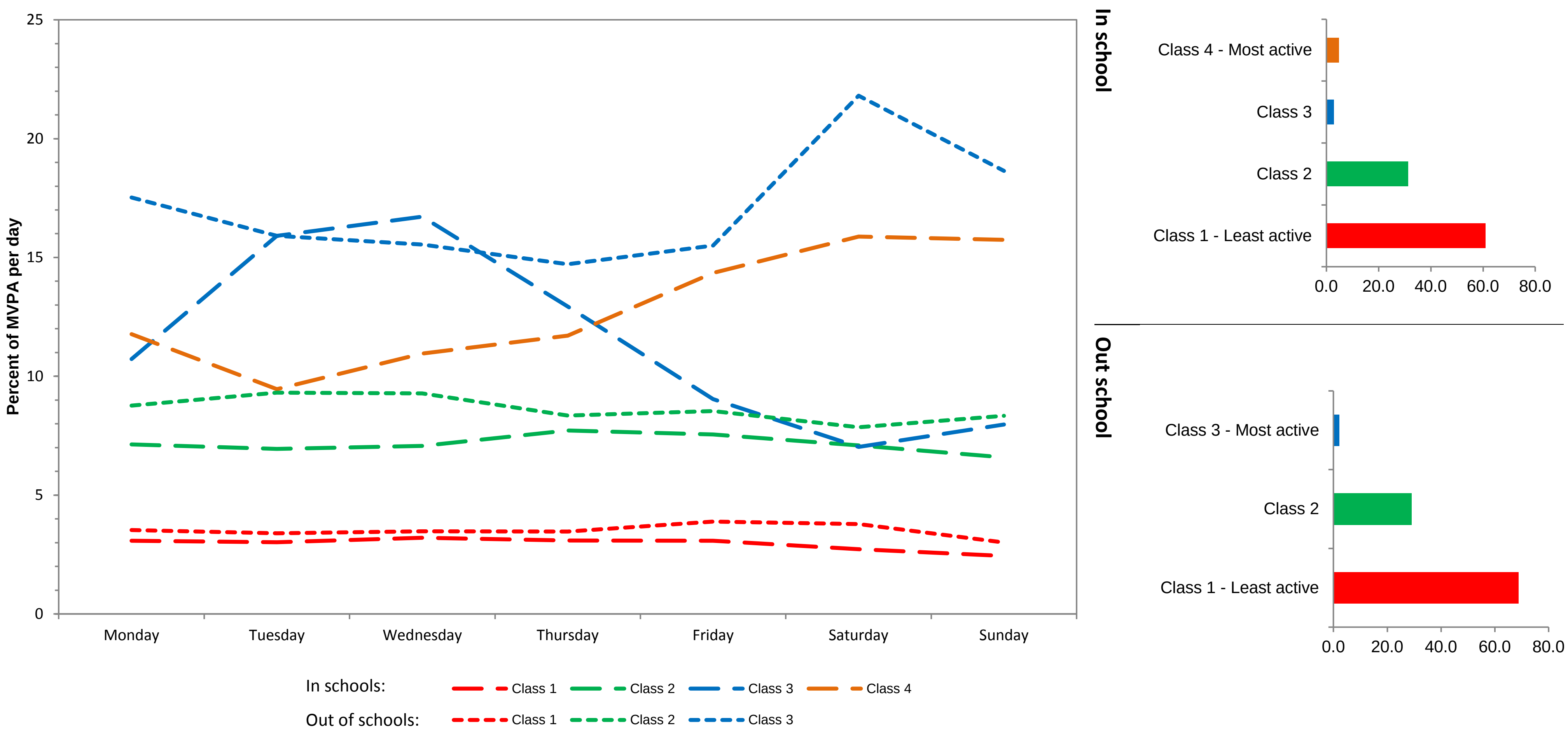

Online Figure 5a: Latent class analysis plotted for weighted percent of vigorous physical activity out of total wearing time per day by age groups; NHANES 2003-2006

**Latent class pattern**	**Weighted %**

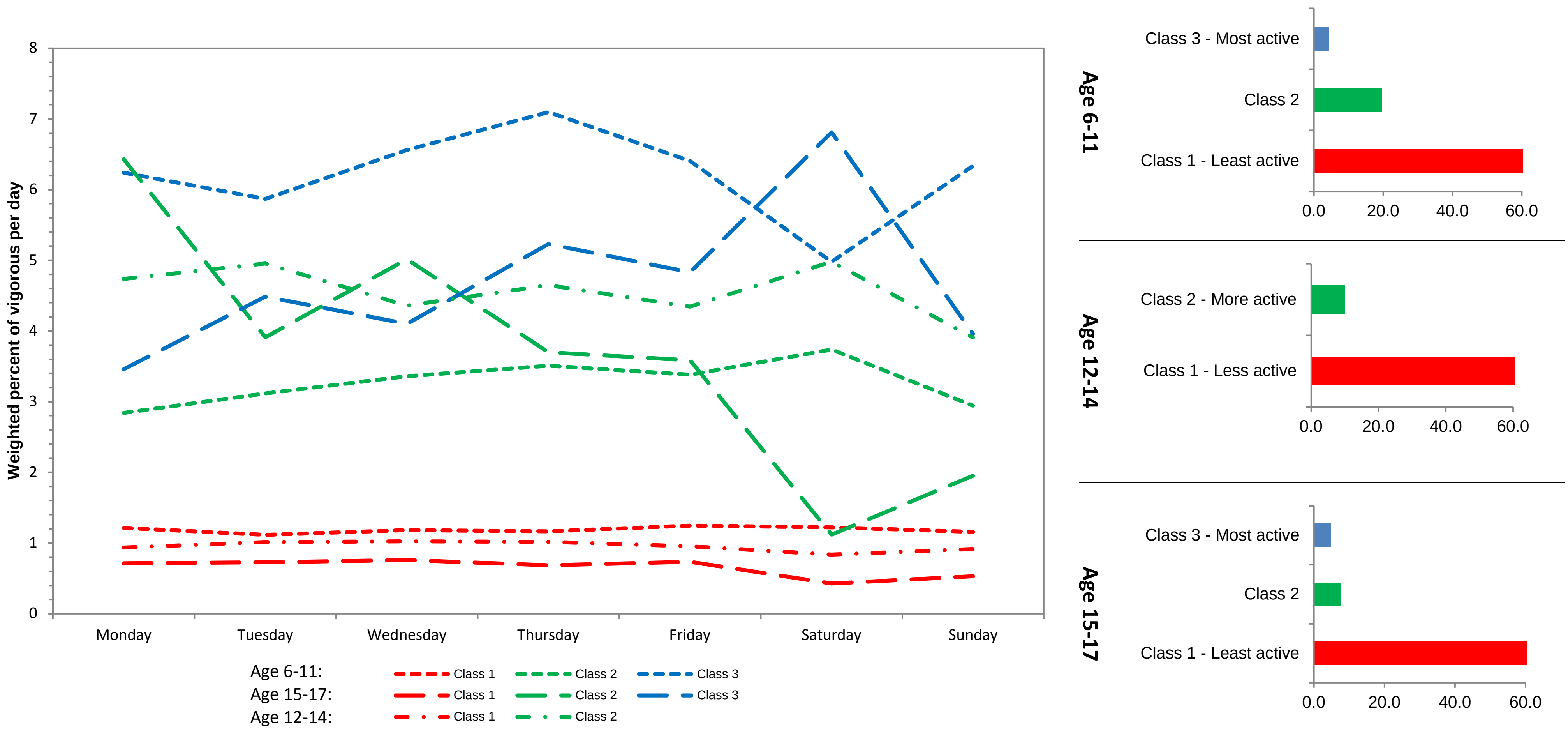

Online Figure 5b: Latent class analysis plotted for weighted percent of vigorous physical activity out of total wearing time per day by gender; NHANES 2003-2006

Latent class pattern **Weighted %**

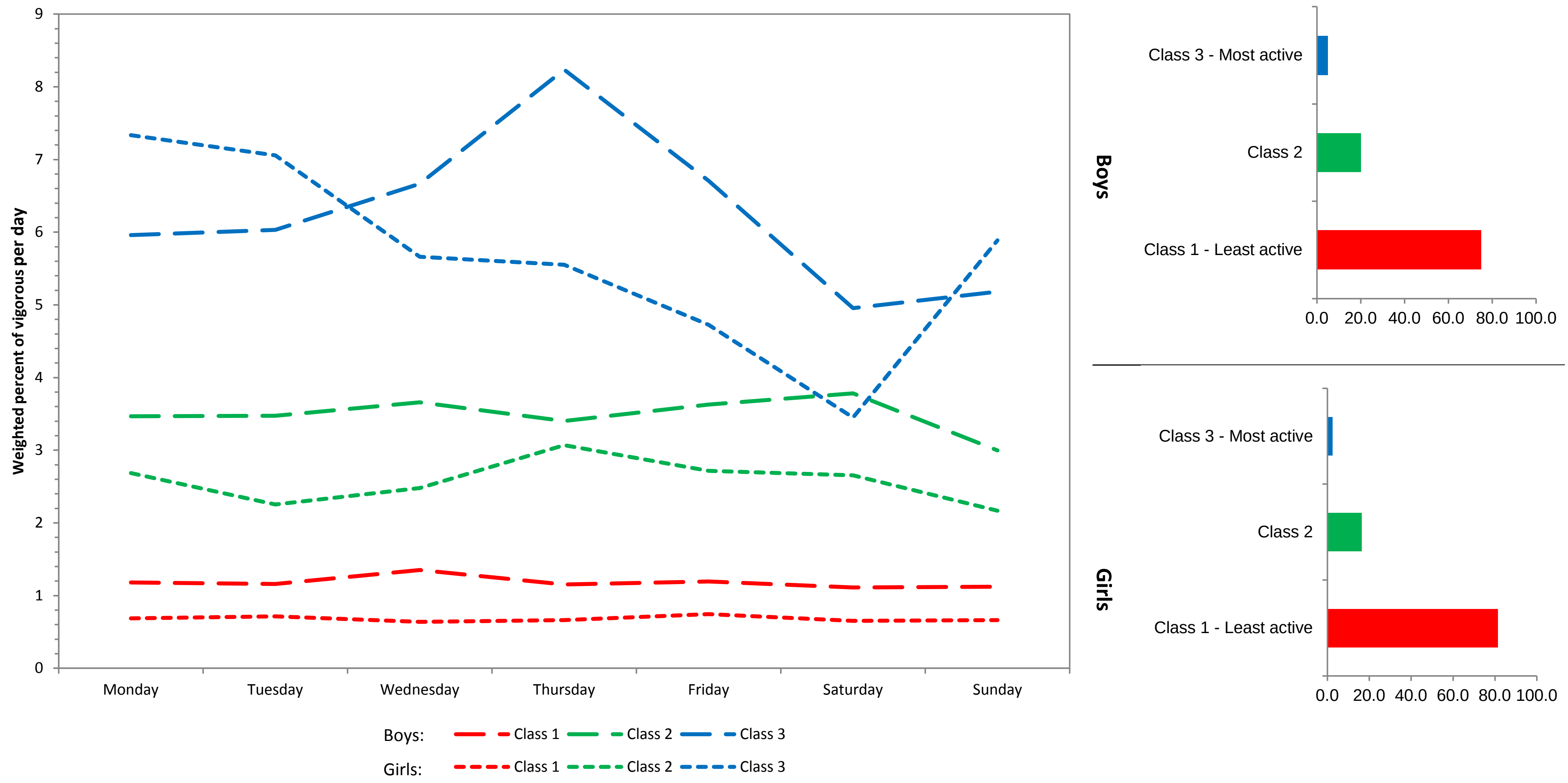

Online Figure 5c: Latent class analysis plotted for weighted percent of vigorous physical activity out of total wearing time per day by in or out of school; NHANES 2003-2006

Latent class pattern	Weighted %

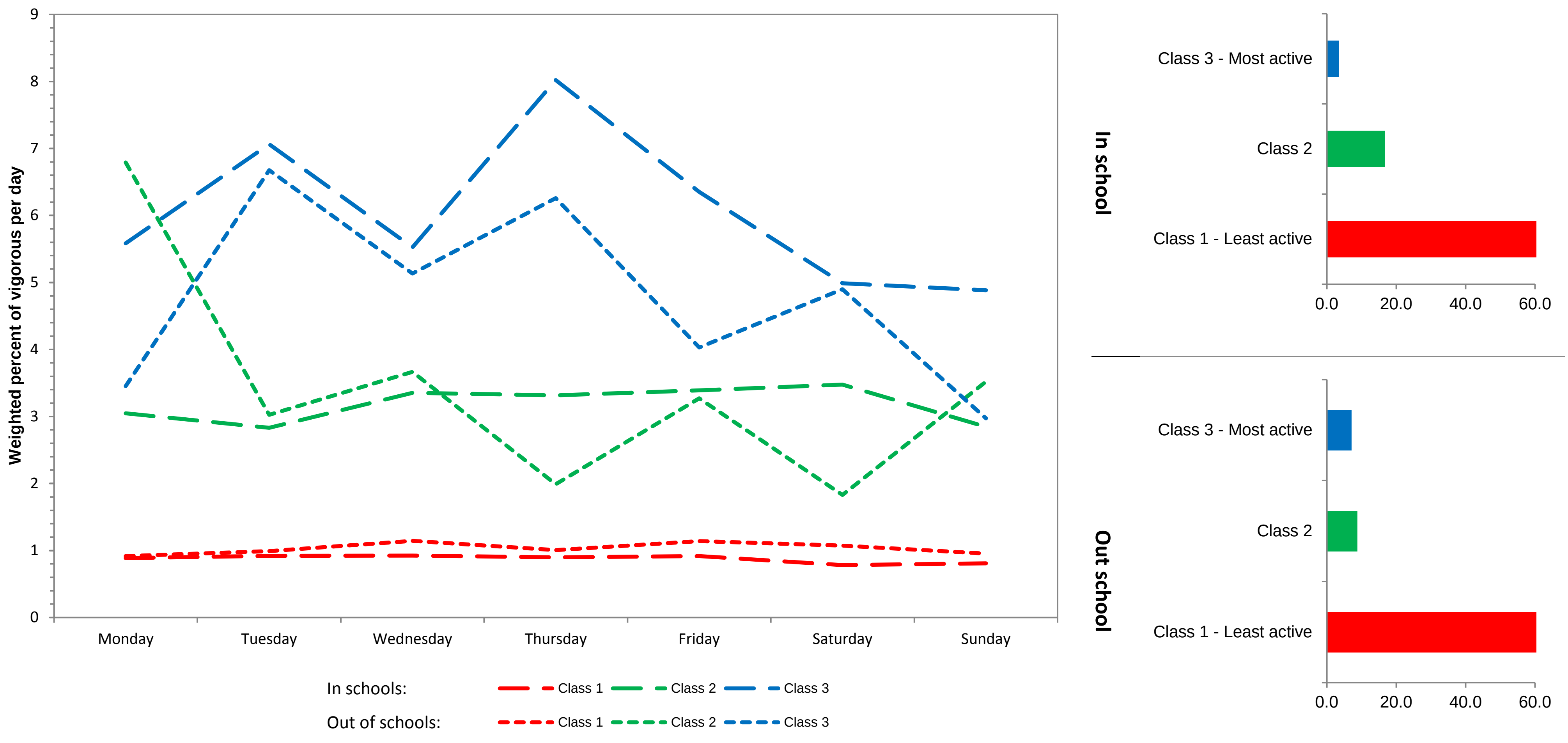

Supplement: Additional file 2: — Latent class analysis plotted for (1) weighted average counts/minute/day and weighted percent of (2) sedentary behavior, (3) light activity, (4) moderate to vigorous physical activity and (5) vigorous activity out of total wearing time per day, by age, gender, and school characteristics, among youth 6–17 years; NHANES 2003–2006. (PDF 144 kb) [file 12966_2016_382_MOESM2_ESM.pdf]
