# Supplementary material for: National youth sedentary behavior and physical activity daily patterns using latent class analysis applied to accelerometry
Source: Int J Behav Nutr Phys Act. 2016 May 3;13:55. doi: 10.1186/s12966-016-0382-x (PMC4855777; doi:10.1186/s12966-016-0382-x)
Supplement: Additional file 3: — Online Table 2: Weighted average physical activity and sedentary behavior in minutes/day by day of the week for each latent class derived from accelerometry, overall and by age, gender, and school characteristics, among youth 6–17 years; NHANES 2003–2006. (PDF 44 kb) [file 12966_2016_382_MOESM3_ESM.pdf]

Online Table 2: Weighted average physical activity and sedentary behavior in minutes/day by day of the week for each latent class derived from accelerometry, overall and by age, gender, and school characteristics, among youth 6-17 years; NHANES 2003-2006

		Weighted Average Minutes/Day						
		By Day of the Week						
	n	Monday	Tuesday	Wednesday	Thursday	Friday	Saturday	Sunday
Latent class: percent of sedentary behavior (0-<100 counts/minute) out of total wearing time per day								
Overall:								
Class 1 - Most sedentary	538	564.3	570.9	572.2	575.3	560.4	497.7	494.8
Class 2	1405	474.8	481.4	469.6	462.6	478.3	411.9	423.4
Class 3	1461	373.9	368.1	369.6	373.3	365.5	338.6	327.1
Class 4 -Least sedentary	594	267.2	273.0	257.4	252.9	275.1	256.9	246.5
By age:								
Age 6-11 years old								
Class 1 - Most sedentary	120	485.3	508.6	488.7	469.8	502.0	384.6	422.4
Class 2	587	402.5	400.1	405.9	409.1	403.0	373.3	366.2
Class 3	640	328.2	320.3	318.3	314.7	330.3	285.1	275.4
Class 4 -Least sedentary	241	231.0	242.1	223.2	220.0	240.6	228.1	223.4
Age 12-14 years old								
Class 1 - Most sedentary	103	601.6	595.0	584.4	607.6	602.9	519.2	518.5
Class 2	463	495.9	512.8	506.5	495.7	493.2	430.8	428.3
Class 3	518	417.0	417.6	406.2	395.7	409.5	366.2	360.2
Class 4 -Least sedentary	163	323.6	301.2	311.5	301.3	300.9	306.1	306.6
Age 15-17 years old								
Class 1 - Most sedentary	204	599.4	597.8	587.3	604.6	561.1	507.4	494.0
Class 2	605	504.5	506.2	490.2	502.4	508.4	451.3	464.6
Class 3	290	402.8	406.3	408.6	398.5	391.9	367.2	382.8
Class 4 -Least sedentary	64	272.3	304.8	287.0	289.7	284.8	382.3	294.7
By gender:								
Boys								
Class 1 - Most sedentary	267	559.9	549.9	553.2	539.8	556.6	507.2	502.8
Class 2	743	461.5	460.7	455.1	455.9	466.9	398.2	412.6
Class 3	741	356.7	349.7	349.0	353.2	355.3	330.5	325.0
Class 4 -Least sedentary	255	250.7	251.0	243.9	234.3	249.9	237.5	236.0
Girls								
Class 1 - Most sedentary	275	565.7	583.4	580.6	589.9	567.8	486.0	490.8
Class 2	693	485.5	498.5	484.7	475.4	479.5	424.2	424.5
Class 3	697	385.5	381.3	381.0	383.8	373.2	347.2	329.8
Class 4 -Least sedentary	327	283.5	291.4	275.3	273.6	293.5	265.5	253.3
By school characteristics:								
In school								
Class 1 - Most sedentary	341	576.2	593.7	589.4	586.9	574.7	502.2	492.9
Class 2	978	501.5	504.0	491.5	486.6	491.7	425.9	436.7

Class 3	1058	406.5	404.4	399.8	401.6	403.0	361.0	352.3
Class 4 -Least sedentary	497	306.8	307.9	297.6	292.5	304.5	275.7	272.8
Out school								
Class 1 - Most sedentary	239	493.0	497.6	480.9	510.1	513.4	456.2	475.1
Class 2	415	363.1	368.2	377.6	372.6	372.5	352.5	360.0
Class 3 -Least sedentary	163	267.5	282.6	249.1	249.2	265.4	259.1	251.6
Latent class: percent of light activity (100-2295 counts/minute) out of total wearing time per day								
Overall:								
Class 1 - Least light activity	508	229.1	218.6	214.9	216.8	255.2	225.0	226.8
Class 2	1379	303.3	303.9	301.9	303.4	320.8	313.2	294.5
Class 3	1572	386.3	386.7	389.2	395.5	402.6	382.5	368.2
Class 4 - Most light activity	539	462.1	464.7	468.4	469.9	470.8	440.3	427.1
By age:								
Age 6-11 years old								
Class 1 - Least light activity	228	304.1	294.0	297.1	291.0	314.2	326.2	298.0
Class 2	906	387.0	387.5	387.7	394.3	405.8	382.8	376.1
Class 3 - Most light activity	454	467.0	460.6	467.8	467.5	469.0	444.2	429.9
Age 12-14 years old								
Class 1 - Least light activity	363	246.4	246.1	240.4	251.8	275.9	252.8	251.3
Class 2	662	345.2	340.0	337.2	339.2	353.0	339.2	311.6
Class 3 - Most light activity	222	384.8	414.2	419.9	427.1	444.8	415.5	390.6
Age 15-17 years old								
Class 1 - Least light activity	409	233.6	219.9	216.7	223.0	258.9	236.7	233.4
Class 2	586	314.1	326.9	322.7	321.7	328.0	316.9	293.2
Class 3 - Most light activity	168	415.4	417.0	437.3	446.7	433.0	377.4	374.9
By gender:								
Boys								
Class 1 - Least light activity	247	228.9	216.1	215.4	214.0	253.8	225.0	224.1
Class 2	706	309.6	311.3	308.5	307.5	321.5	320.6	302.0
Class 3	808	395.4	394.9	393.5	401.0	405.3	383.3	375.0
Class 4 - Most light activity	245	471.8	481.0	474.2	472.2	478.0	439.2	428.7
Girls								
Class 1 - Least light activity	252	228.7	217.0	209.9	220.1	253.8	227.6	228.0
Class 2	678	298.2	294.9	295.4	299.1	321.4	302.2	288.7
Class 3	759	373.5	375.0	382.7	388.3	397.1	380.9	360.4
Class 4 - Most light activity	303	449.0	449.9	459.0	462.1	464.6	439.7	418.9
By school characteristics:								
In school								
Class 1 - Least light activity	468	233.6	229.5	217.9	225.1	255.2	229.2	234.6
Class 2	1061	310.4	304.6	307.4	309.5	328.4	320.6	298.8
Class 3	1042	385.8	385.3	391.0	398.2	405.1	385.0	367.8
Class 4 - Most light activity	303	454.3	450.9	464.3	462.2	460.8	420.2	422.0
Out school								

Class 1 - Least light activity	266	252.5	259.1	250.5	251.3	272.5	267.8	248.7
Class 2	414	362.8	371.3	369.7	371.5	375.1	362.5	343.5
Class 3 - Most light activity	137	455.2	468.7	455.7	456.1	464.9	467.8	439.1
Latent class: percent of moderate to vigorous physical activity (>=2296 counts/minute) out of total wearing time per day								
Overall:								
Class 1 - Least active	2430	26.6	25.3	27.1	26.1	27.4	23.3	20.1
Class 2	1243	62.5	60.1	60.1	63.3	66.5	59.2	54.9
Class 3	162	85.3	126.5	125.5	90.9	69.9	53.0	52.0
Class 4 - Most active	163	107.3	90.9	97.0	113.0	124.7	124.3	121.6
By age:								
Age 6-11 years old								
Class 1 - Least active	886	33.3	32.2	33.5	33.0	35.5	33.6	29.5
Class 2	586	65.5	65.3	68.6	70.0	70.9	65.7	61.4
Class 3 - Most active	116	104.6	99.0	113.5	110.8	120.5	120.8	119.0
Age 12-14 years old								
Class 1 - Least active	783	24.3	24.2	25.2	24.7	26.0	19.5	16.6
Class 2	348	57.4	60.8	54.3	57.5	57.6	53.8	53.0
Class 3 - Most active	116	113.2	98.8	102.3	109.6	99.6	88.0	71.7
Age 15-17 years old								
Class 1 - Least active	903	24.1	23.4	24.6	22.7	25.0	17.9	14.0
Class 2	195	91.7	65.0	78.7	78.0	72.3	48.8	32.0
Class 3 - Most active	65	37.5	92.7	58.3	56.5	60.6	29.4	110.8
By gender:								
Boys								
Class 1 - Least active	1091	33.7	30.5	35.5	32.5	33.5	30.8	25.8
Class 2	679	70.9	66.2	67.6	68.8	74.3	66.6	64.0
Class 3	123	79.4	132.7	127.1	97.6	70.1	49.1	34.8
Class 4 - Most active	113	106.3	93.0	100.0	115.4	134.2	133.1	120.4
Girls								
Class 1 - Least active	1437	22.5	23.5	22.3	22.6	25.3	18.9	16.8
Class 2	514	56.3	54.0	55.4	61.5	60.8	54.1	49.0
Class 3 - Most active	41	108.4	114.0	110.1	87.8	74.3	79.7	116.4
By school characteristics:								
In school								
Class 1 - Least active	1739	25.6	24.8	26.3	25.7	26.2	20.7	17.9
Class 2	892	59.0	57.5	57.9	62.8	63.1	54.2	49.2
Class 3	108	89.0	133.7	135.1	104.1	76.2	55.3	60.2
Class 4 - Most active	135	96.1	77.5	89.2	97.5	114.8	121.3	110.7
Out school								
Class 1 - Least active	559	26.9	26.7	26.8	26.6	30.4	28.1	21.9
Class 2	234	71.5	75.1	71.6	66.0	68.5	59.3	62.2
Class 3 - Most active	24	134.8	121.3	109.0	111.8	108.9	148.5	119.9

Latent class: percent of vigorous physical activity (>=4012 counts/minute) out of total wearing time per day								
Overall:								
Class 1 - Least active	3023	7.4	7.3	7.5	7.2	7.8	6.6	6.4
Class 2	743	24.8	23.3	25.7	25.1	25.3	23.4	18.8
Class 3 - Most active	232	49.0	51.7	47.7	54.6	50.6	37.4	38.9
By age:								
Age 6-11 years old								
Class 1 - Least active	1187	9.7	8.9	9.4	9.3	10.3	9.2	8.6
Class 2	314	23.0	25.2	26.2	28.2	27.5	27.9	21.2
Class 3 - Most active	87	51.0	47.9	53.6	58.1	53.3	39.5	46.8
Age 12-14 years old								
Class 1 - Less active	1082	7.8	8.4	8.4	8.4	8.1	6.4	6.7
Class 2 - More active	165	40.1	41.1	35.4	37.6	35.9	37.5	28.6
Age 15-17 years old								
Class 1 - Least active	992	6.0	6.0	6.4	5.8	6.1	3.2	4.0
Class 2	104	51.1	33.0	39.2	29.7	29.8	8.8	14.9
Class 3 - Most active	67	31.8	37.0	34.1	43.8	39.4	57.0	28.9
By gender:								
Boys								
Class 1 - Least active	1408	9.7	9.5	11.0	9.4	10.1	8.5	8.4
Class 2	464	28.9	28.6	29.3	27.5	30.1	28.2	22.3
Class 3 - Most active	134	50.3	50.5	53.4	65.7	56.2	38.8	37.3
Girls								
Class 1 - Least active	1674	5.5	5.9	5.2	5.4	6.1	4.9	4.8
Class 2	272	21.5	17.9	19.6	25.1	21.7	20.7	15.6
Class 3 - Most active	46	55.9	55.9	44.8	46.8	37.7	27.9	43.7
By school characteristics:								
In school								
Class 1 - Least active	2263	7.3	7.6	7.6	7.4	7.8	6.0	6.1
Class 2	482	25.0	23.5	27.6	27.1	28.3	26.3	20.9
Class 3 - Most active	129	46.8	59.4	45.9	65.6	53.5	39.8	36.6
Out school								
Class 1 - Least active	671	7.3	8.0	8.9	7.9	9.0	8.0	7.1
Class 2	83	53.2	25.0	27.0	15.7	25.5	13.5	24.1
Class 3 - Most active	63	29.2	50.6	38.3	49.7	31.7	37.7	21.4
